# What is the level of evidence for the amnestic effects of sedatives in pediatric patients? A systematic review and meta-analyses

**DOI:** 10.1371/journal.pone.0180248

**Published:** 2017-07-07

**Authors:** Karolline Alves Viana, Anelise Daher, Lucianne Cople Maia, Paulo Sucasas Costa, Carolina de Castro Martins, Saul Martins Paiva, Luciane Rezende Costa

**Affiliations:** 1 Programa de Pós-graduação em Odontologia, Universidade Federal de Goiás (UFG), Goiânia, Goiás (GO), Brazil; 2 Departmento de Odontopediatria e Ortodontia, Universidade Federal do Rio de Janeiro, Rio de Janeiro, Rio de Janeiro, Brazil; 3 Departmento de Pediatria, Faculdade de Medicina, UFG, Goiânia, GO, Brazil; 4 Departamento de Odontopediatria e Ortodontia, Faculdade de Odontologia, Universidade Federal de Minas Gerais, Belo Horizonte, Minas Gerais, Brazil; 5 Departamento de Saúde Oral, UFG, Goiânia, GO, Brazil; Memorial Sloan Kettering Cancer Center, UNITED STATES

## Abstract

**Background:**

Studies have suggested that benzodiazepines are amnestic drug par excellence, but when taken together, what level of evidence do they generate? Are other sedatives as amnestic as benzodiazepines? The aim of this study was to assess the level of scientific evidence for the amnestic effect of sedatives in pediatric patients who undergo health procedures.

**Methods:**

The literature was searched to identify randomized controlled trials that evaluated anterograde and retrograde amnesia in 1-19-year-olds who received sedative drugs during health procedures. Electronic databases, including PubMed, Scopus and Cochrane Library besides clinical trial registries and grey literature were searched. Two independent reviewers performed data extraction and risk of bias assessment using the Cochrane Collaboration's Tool. The meta-analyses were performed by calculating relative risk (RR) to 95% confidence intervals (CI). The quality of the evidence was assessed using Grading of Recommendations Assessment, Development and Evaluation approach.

**Results:**

Fifty-four studies were included (4,168 participants). A higher occurrence of anterograde amnesia was observed when benzodiazepines, the most well-studied sedatives (n = 47), were used than when placebo was used (n = 12) (RR = 3.10; 95% CI: 2.30–4.19, *P*<0.001; I^2^ = 14%), with a moderate level of evidence. Higher doses of alpha2-adrenergic agonists (clonidine/dexmedetomidine) produced more anterograde amnesia than lower doses (n = 2) (RR = 1.83; 95% CI: 1.03–3.25; *P =* 0.038; I^2^ = 0%), with a low level of evidence; benzodiazepines’ amnestic effects were not dose-dependent (n = 3) (RR = 1.54; 95% CI: 0.96–2.49; *P =* 0.07; I^2^ = 12%) but the evidence was low. A qualitative analysis showed that retrograde amnesia did not occur in 8 out of 10 studies.

**Conclusions:**

In children, moderate evidence support that benzodiazepines induce anterograde amnesia, whereas the evidence for other sedatives is weak and based on isolated and small studies. Further clinical trials focused on the amnesia associated with non-benzodiazepine sedatives are therefore needed.

**Trial registration:**

PROSPERO CRD42015017559.

## Introduction

Health procedures can lead to fear, anxiety and behavior management problems in children and adolescents. Pediatric patients can benefit from sedation, a pharmacological approach that aims to control anxiety and behavior, reduce physical discomfort, promote patient safety, and minimize the chance of psychological trauma by maximizing the potential for amnesia [1].

It is widely accepted that some responsiveness is expected during sedation. Specifically, patients may respond normally or purposefully to a stimulus [2]. However, sedation can fail in some situations, meaning that the patient has unwanted degrees of uncooperative behavior that requires intervention, such as protective stabilization. Over the past five years, it has been shown that the success rate of sedation in children and adolescents in a medical or a dental setting ranges from 26.7% [3] to 96.2% [4]. Thus, if pediatric patients are responsive and/or show a distressed reaction while sedated, the question of whether they remember perioperative events is an important one. In the practice of anesthesia, amnesia is a therapeutically desirable effect [5] that is considered one of the key pillars of the triad of anesthesia [6]. Remembering an aversive stimulus can lead to the development of psychological trauma [7]. In fact, it has been demonstrated that memories of distressing events play a significant role in the development of dental phobias [8]. Moreover, children that experience traumatic clinical procedures are expected to display negative behaviors in future dental appointments [9,10].

Thus, amnesia is an important component of sedation for pediatric patients who will undergo uncomfortable healthcare procedures. Some sedatives can impair memory temporarily either directly, via drug interference with memory process (drug-induced amnesia), or indirectly, by impairing attention and arousal secondary to their sedative effects [11,12]. A wide variety of drugs have been investigated to explore their effects on either anterograde or retrograde amnesia, which refer to the inability to consciously recall information presented after or before drug intake, respectively [12]. Although the memory effects of sedatives have been reported in children, it is still unclear to what extent amnesia is present in clinical practice [11, 13–19]. The degree of amnesia can differ according to the drug, the measurement used to study memory, and the characteristics of the participants [11]. While in some studies, all participants presented complete anterograde amnesia for procedure [13–17], in other investigations, only a few patients failed to remember perioperative events [18,19]. New knowledge regarding the potential of different sedatives to produce amnesia would therefore be pivotal to the decision-making process that takes place when choosing a sedative regimen for a pediatric patient because young children can show uncooperative behavior even when sedated.

Interestingly, the outcomes of randomized clinical trials on sedation related amnesia have not been pooled for systematic analyses. Hence, the aim of this systematic review was to highlight the level of evidence for the amnestic effects of sedatives in pediatric patients who undergo health procedures.

## Methods

### Protocol and registration

The methods used to perform this systematic review were previously reported as a study protocol (S1 Protocol) [20]. This study is registered in the PROSPERO database under the number CRD4201501755. The present report follows the recommendations of the Preferred Reporting Items for Systematic Reviews and Meta-Analysis (PRISMA; S1 Appendix) [21].

### Eligibility criteria

The eligibility criteria were chosen using the PICOS (population, intervention, comparator, outcomes and study design) strategy: 1) population: pediatric patients aged 1–19 years old who received sedative drugs as a premedication or as agents for procedural sedation; 2) intervention: any sedative regimen administered by a health professional in an outpatient setting or operating room; 3) comparator: placebo, variations of the same sedative regimen (i.e., dosage, route and timing of administration) or an alternative sedation regimen; 4) outcome: anterograde amnesia (primary endpoint) was defined as the loss of memories of events that occurred after sedative administration, while retrograde amnesia (secondary endpoint) was defined as the loss of memories of events that occurred before sedative administration; and 5) study design: randomized controlled trials (RCT).

Studies involving patients with cognitive or neurological impairments in addition to RCTs that reported pooled and undifferentiated data on both adolescents and adults were excluded. No restrictions were placed on the date of publication, the publication status or the publication language.

### Search strategy and information sources

The search strategy was developed under the guidance of a librarian. Controlled vocabulary, synonyms, related terms and free terms related to children/adolescents, sedatives and memory were combined and searched without filters or limits. The search strategy followed the syntax rules of each database (S1 Table). One reviewer (KAV) performed the electronic searches between September 26^th^ and October 1^st^ 2015, and another (AD) reviewed them to check for errors.

To identify trials eligible for inclusion in this review, the searches were performed in the electronic bibliography databases of the Public Medical Literature Analysis and Retrieval System Online (PubMed), Scopus, The Cochrane Library, the Latin American and Caribbean Health Sciences Literature database (LILACS), the Brazilian Library in Dentistry (BBO), the Cumulative Index to Nursing and Allied Health Literature (CINAHL), the Web of Science, the Excerpta Medica Database (EMBASE) and PsycINFO. Additionally, the grey literature was searched in the OpenGrey, "ProQuest dissertations and Theses full text" and *Periódicos Capes*. Theses databases through the Brazilian agency Coordination for the Improvement of Higher Education Personnel (*Coordenação de Aperfeiçoamento de Pessoal de Nível Superior–CAPES*). To locate unpublished and ongoing trials, the following trial registries were searched: Current Controlled Trials, the US National Institutes of Health, the Brazilian Clinical Trials Registry (*Registro Brasileiro de Ensaios Clínicos–ReBEC*) and the UK National Institute for Health and Care Excellence.

### Study selection and data collection process

Study selection was performed in duplicate by two independent and calibrated reviewers (KAV and AD). First, duplicate references were removed using the software program EndNote^®^ (EndNote X7, Thomson Reuters, New York, USA). Next, as a training and calibration exercise, the independent reviewers applied the eligibility criteria to 10% of the titles/abstracts of the retrieved studies, and inter-examiner agreement was calculated. This exercise was repeated until there was almost perfect agreement, which was achieved at a Kappa coefficient ≥ 0.8 [22] (Kappa = 0.81; CI 95% 0.70–0.92). Disagreements were resolved by consensus using the supervision of a gold standard (LRC). Finally, each independent reviewer selected the remaining studies by their titles and abstracts based on the eligibility criteria.

The full-text of the potentially eligible studies, which were those that at least one reviewer regarded as having met the inclusion criteria, were read and judged based on the eligibility criteria. Discrepancies were solved by a third reviewer (LRC).

Two trained independent reviewers (KAV and AD) performed data collection in duplicate. A standardized data collection form was developed and pilot-tested using a randomized sample of three included trials. This form was refined accordingly. The following data were recorded for each included study: article identification characteristics; study design; patient information; type of intervention and comparisons used; whether the sedative was used as procedural sedation or premedication; setting (i.e., outpatient setting or operating room) and treatment performed; type of memory or amnesia; type of outcome measurement; statistical techniques used and results of the study analysis. Disagreements were solved by consensus.

### Risk of bias in individual studies

The risk of bias assessment in the included studies was evaluated using the Cochrane Collaboration’s Tool for Assessing Risk of Bias in Randomized Trials [23]. The assessment criteria contained seven specific domains: random sequence generation, allocation concealment, blinding of participants and personnel, blinding of outcome assessment, incomplete outcome data, selective reporting and other bias.

For each domain, the risk of bias was graded as high, low or unclear based on criteria described in the Cochrane Handbook for Systematic reviews of Interventions 5.1.0 (http://handbook.cochrane.org) [24]. Only four out of the seven domains were considered key domains for assessing the risk of bias of the studies: random sequence generation, allocation concealment, blinding of participants and personnel, and blinding of outcome assessment. Studies were considered to be at 'low' risk of bias when there was 'low' risk of bias in all of these key domains. When the study was judged as 'high' or 'unclear' in one of these key domains, it was considered, respectively, at 'high' or 'unclear' risk of bias.

Any disagreements between the reviewers were solved by consensus or by consulting a third reviewer (LRC).

### Synthesis of the results

A narrative summary of study characteristics is provided in the text and presented in the tables. Outcome results/conclusions are shown as numerical data and/or statistical results when this information were available in studies. Differences are reported as statistically significant if the trial reported *P*<0.05.

Included studies with low and uncertain risk of bias were analyzed in relation to clinical and methodological heterogeneity to determine whether a meta-analysis could be performed. Among the studies that could be meta-analyzed, statistical heterogeneity was evaluated using chi-squared tests and Higgins and Thompson’s I^2^. When heterogeneity was substantial (I^2^ >50%, P<0.1) [24], a sensitivity analysis was performed to explore the influence of each study on the pooled data. To summarize the data obtained from each study, the relative risk (RR) was calculated with a 95% confidence interval (CI). A random-effects model was used. All analyses were conducted using The Comprehensive Meta-Analysis software program, version 3 (Biostat, Inc., Englewood, USA). Publication bias was evaluated by visually inspecting the funnel plot and using Egger’s test when at least 10 studies were included in meta-analysis. For other studies, bias was assessed by verifying the presence of both significant and non-significant outcomes.

The quality of the included evidence was evaluated using the approach described in Grading of Recommendations Assessment, Development and Evaluation (GRADE) [25]. The assessments were based on study design, risk of bias, presence of imprecision, inconsistency, indirectness and publication bias.

## Results

### Study selection

**Fig 1:**
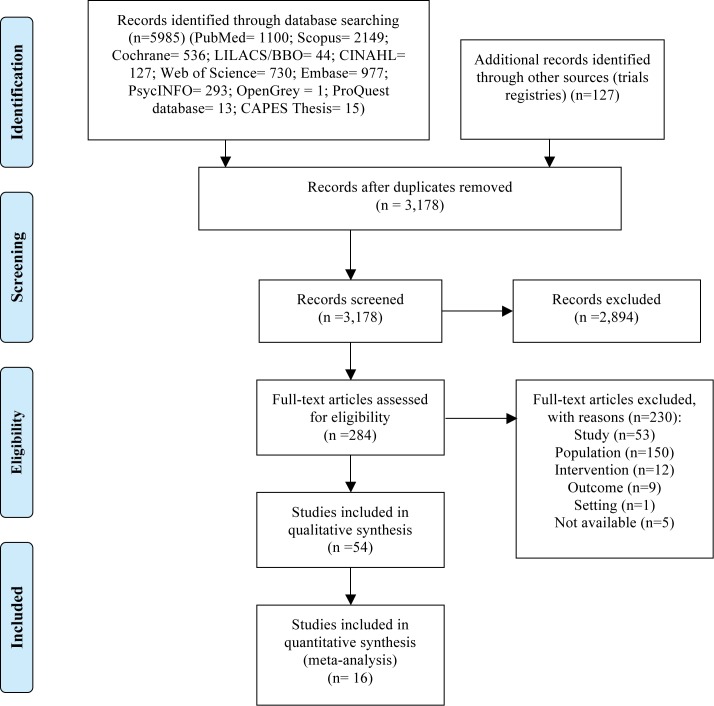
Flow diagram of literature search.

A total of 6,112 studies were identified in the search. After duplicates were removed, 3,178 studies remained. Screening of the titles and abstracts resulted in the exclusion of 2,894 records (Fig 1). The main reasons for exclusion were non-related subjects (n = 2,550), non-RCT study (n = 208) and other setting (n = 89). Of the 284 potentially eligible studies, 54 met the inclusion criteria and were included in the systematic review. Among these, the data from 16 studies were meta-analyzed: 12 were pooled to compare benzodiazepines vs placebo, 3 to compare dosages of benzodiazepines and 2 to compare dosages of alpha2-adrenergic agonists. One study was included in two different meta-analyses.

### Characteristics of included articles

The demographic (location/year) and intervention (sedative use/procedure/setting) characteristics of all the included articles were summarized in Table 1, whereas detailed characteristics of each study were stated in S2 to S6 Tables.

**Table 1:** Characteristics of included articles.

Characteristics	N	%
Location		
United States	19	35.2
United Kingdom	9	16.7
Others	26	48.1
Time since publication		
≥ 10 years	45	83.3
< 10 years	9	16.7
Sedative use		
Procedural sedation	26	48.1
Premedication	28	51.9
Procedure		
Dental	17	31.5
Medical	34	63.0
Dental or medical	2	3.7
Not mentioned	1	1.8
Setting		
Operating room	27	50.0
Outpatient	27	50.0

The included studies were performed in 20 different countries, and most of them (n = 19, 35.2%) were performed in the United States [7,14–16, 26–40]. The studies were published from 1969 to 2015.

The number of patients included in the studies ranged from 12 [32] to 260 [34] (total = 4,168). The age range varied from 0 months to 18 years, but the outcome ‘memory’ was not evaluated in younger children (<1 year).

In almost half of studies, sedatives were used as agents for procedural sedation [13–15,19,27–30,32–34,36–39, 41–51] instead of premedication, and half of the trials were performed in an outpatient setting. In a great deal of studies, only medical procedures were performed [7,13–19,26,28,30,31,33–35,38–40,46,47,52–65].

A wide variety of sedative regimens were compared. The studies differed according to the drug used and its dose and route, and there were additional differences in the comparator arm. Benzodiazepines, used solely or in combination, were the most well-studied drugs: only 7 out 54 trials did not include a benzodiazepine group [13,17,30,38,46,49,65]. Drugs currently used in clinical practice are reported in the following studies: midazolam was investigated in 33 trials [7, 14–16, 19, 27, 28, 31, 33, 35, 39–45, 47, 48, 50–54, 59, 61, 63, 66–71], nitrous oxide in seven [26, 30, 34, 42, 46, 50, 51], ketamine in eight [13–17, 26, 34, 37, 44, 49, 70], dexmedetomidine in one [49] and propofol in four [13, 14, 46, 47]. Besides, all drugs investigated in 23 studies were the ones currently used: 19 involved midazolam [7, 19, 27, 31, 33, 35, 40, 41, 50–52, 54, 61, 63, 66–70], three nitrous oxide [46, 50, 51], four ketamine [13, 17, 49, 70], one dexmedetomidine [49] and two propofol [13,46].

Anterograde amnesia was evaluated in all studies, whereas retrograde amnesia was assessed in only 10 studies [7,33,35,37,39,40,52,54,60,66]. Amnesia was evaluated mainly by measuring patient recall of pictures/toys and events, except for the six trials [13,41,16,19,38,42]. The results that were reported in all of the trials were predominantly dichotomous (e.g. the presence/absence of amnesia/recall).

### Risk of bias in included studies

**Fig 2:**
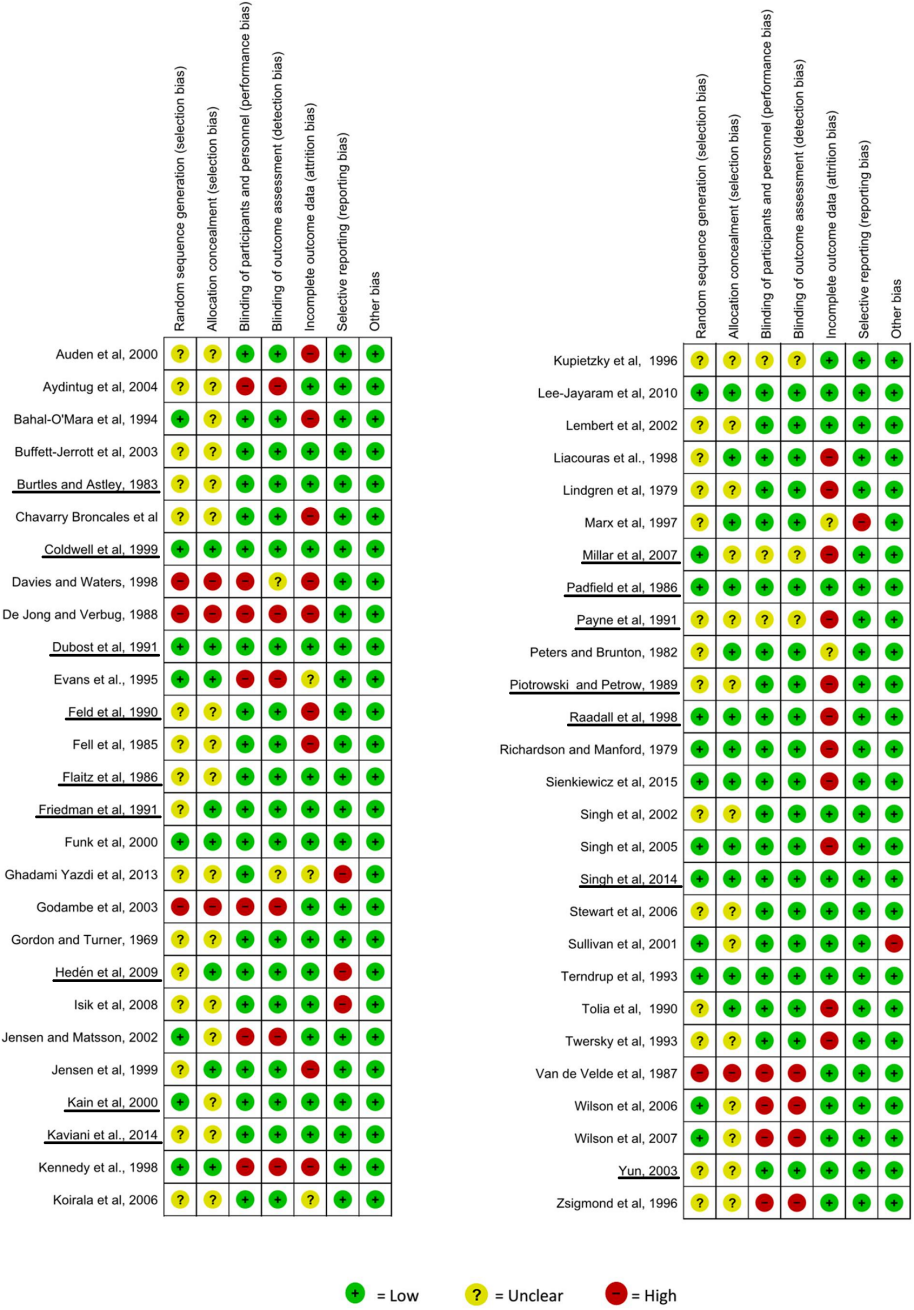
Risk of bias assessment of the included studies (The Cochrane Collaboration tool for assessing risk of bias).

The risk of bias assessment performed on the included studies is presented in Fig 2. Studies included in meta-analyses were underlined in this figure. Most of the studies were found to have unclear risk (n = 32; 59.3%), 11 had low risk, and 9 had high risk.

Considering the key domains, in the majority of trials (n = 30, 55.6%), the method used for sequence generation was unclear, as was the reporting of the allocation concealment. The domain ‘blinding of participants and health care providers’ was judged ‘low’ in 40 full text articles (74.1%), and a similar result was found for the domain ‘blinding of outcome assessment’ (n = 39; 72.2%).

Regarding other domains, most studies (n = 29; 53.7%) had low risk for the domain 'incomplete outcome data assessment'. Nearly all studies were considered to have a low risk of bias with regard for selective reporting (n = 50, 92.6%) and other biases (n = 53, 98.1%).

### Evidence synthesis

Because a wide variety of sedative regimens were investigated in the included studies, we categorized the trials into five comparison topics with regard for the outcome ‘anterograde amnesia’: 1. single benzodiazepine versus placebo (S2 Table), 2. single benzodiazepine versus single benzodiazepine (S3 Table), 3. single benzodiazepine versus non-benzodiazepine drug (S4 Table), 4. benzodiazepine in combination with other drugs versus any sedative (S5 Table), and 5. non-benzodiazepine drug versus non-benzodiazepine drug (S6 Table). We attempted to compare sedatives with placebo; where this was not possible, we considered benzodiazepines as standard amnestic drugs.

Trials that evaluated multiple comparisons are presented only once in the tables according to the sequence of categorizations listed above. However, for evidence synthesis and to assess the quality of the presented evidence (Table 2), trials were considered to belong to more than one topic when needed, and in these cases, we took into account only the data related to the drugs being compared. In this systematic review, we excluded the trials’ participants who did not participate in the memory assessment; so, the number of individuals was adjusted to perform meta-analyses as well as in the tables’ column "outcome result/conclusion". No differences were found in the following comparisons when the studies were pooled according to the type of procedure performed (medical or dental) and the setting (outpatient or operating room).

**Table 2:** Quality of evidence on sedatives’ amnestic effects.

Quality assessment							Quantitative assessment	Quality
Number of studies	Study design	Risk of bias	Inconsistency	Indirectness	Imprecision	Other observations		
Anterograde amnesia—benzodiazepines versus placebo								
17 ^1^	RCT	Not serious	Not serious	Not serious	Not serious	Publication bias strongly suspected	Number of patients: sedative 223/418 (53.3%); placebo 50/328 (15.2%)	MODERATE
							Relative effect (95% CI): **RR 3.111** (2.288 to 4.231)	
							Absolute effect (95% CI): **322 more per 1.000**^2^ (from 196 more to 493 more)	
Anterograde amnesia—among benzodiazepines								
17	RCT	Not serious	Not serious	Not serious	Very serious^3^	None		LOW
Anterograde amnesia—benzodiazepines versus non-benzodiazepine sedatives								
13	RCT	Not serious	Not serious	Not serious	Very serious^3^	None		LOW
Anterograde amnesia—benzodiazepines in combination with other drugs versus any sedative								
11	RCT	Not serious	Not serious	Not serious	Very serious^3^	None		LOW
Anterograde amnesia—non-benzodiazepine drugs versus non-benzodiazepine drugs								
8	RCT	Not serious	Not serious	Not serious	Very serious^3^	None		LOW
Retrograde amnesia								
10	RCT	Not serious	Not serious	Not serious	Very serious^3^	None		LOW

RCT: Randomized trials; CI: Confidence interval; RR: Risk ratio;

1. Although 17 studies compared benzodiazepines vs placebo, it was not possible to synthesize effect data for all of them, thus numerical data (number of patients and effect) are related to the 12 trials included in meta-analysis;

2. Amnestic effects may be associated with 322 more anterograde amnesia in 1000 patients sedated with benzodiazepines;

3. Several studies with small sample size and small number of events were found, which increases imprecision.

#### Anterograde amnesia

1. Single benzodiazepine versus placebo

Benzodiazepines used alone were compared to placebos in 17 studies [7,18,19,31–33,35,36,40,52,58,59,63, 64,66,68,69], twelve of which were included in the meta-analysis. The remaining trials were excluded because it was impossible to group the data [40,52,63] or there was an overall high risk of bias [64] or after the sensitivity analysis [35]. In the last case, when the study was removed, heterogeneity decreased from 91% to 14%.

**Fig 3:**
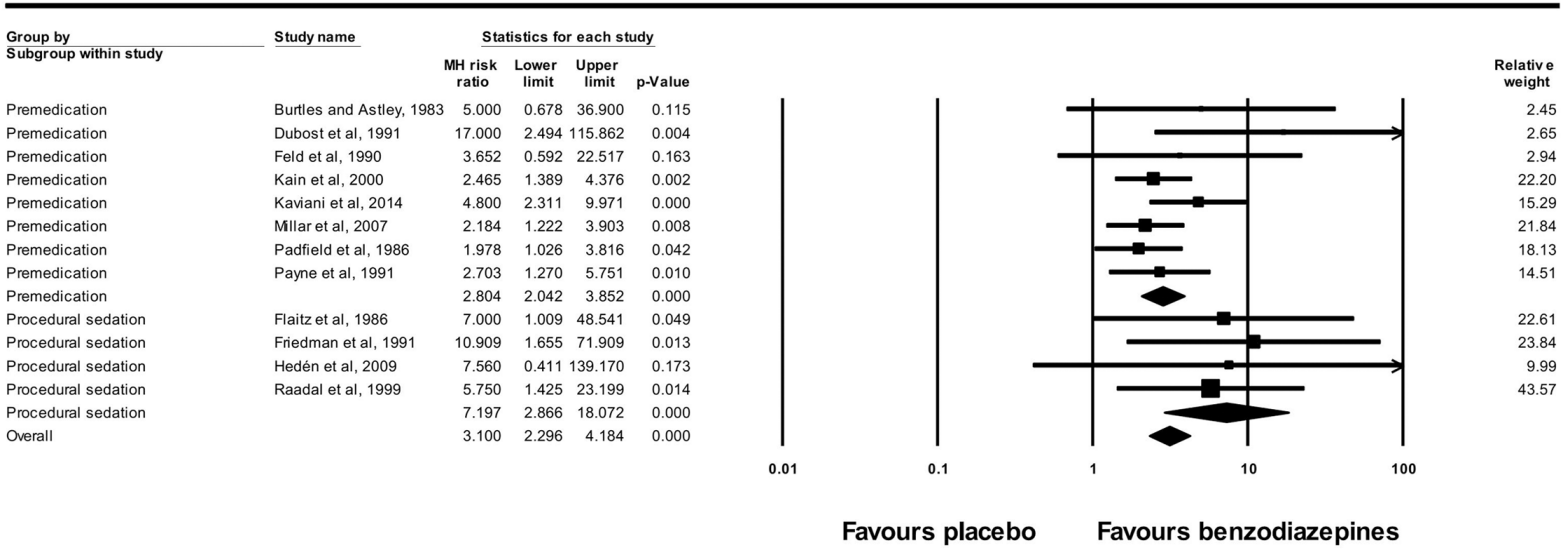
Forest plot of the meta-analysis performed to compare amnestic effects between benzodiazepines and placebos analyzed by subgroups. Premedication: RR = 2.80; 95% CI: 2.04–3.85, *P*<0.001; Procedural sedation: RR = 7.20; 95% CI: 2.87–18.07, *P*<0.001; Overall: RR = 3.10; 95% CI: 2.30–4.18, *P*<0.001. Heterogeneity: premedication I^2^ = 16%, *P* = 0.30; procedural sedation I^2^ = 0%, *P* = 0.96; overall I^2^ = 14%, *P* = 0.30.

The meta-analysis of 12 studies included 746 pediatric patients and revealed that patients who received benzodiazepines displayed stronger anterograde amnesia (223/418) than the patients who received placebo (50/328) (RR = 3.10; 95% CI: 2.30–4.18; *P*<0.001; I^2^ = 14%). This association remains in the analysis of subgroups, when considering sedative used as premedication (RR = 2.80; 95% CI: 2.04–3.85, P<0.001; I^2^ = 16%,) or as agents for procedural sedation (RR = 7.20; 95% CI: 2.87–18.07, P<0.001; I^2^ = 0%) (Fig 3). The benzodiazepines included in the studies were midazolam, lorazepam, diazepam, temazepam and triazolam.

**Fig 4:**
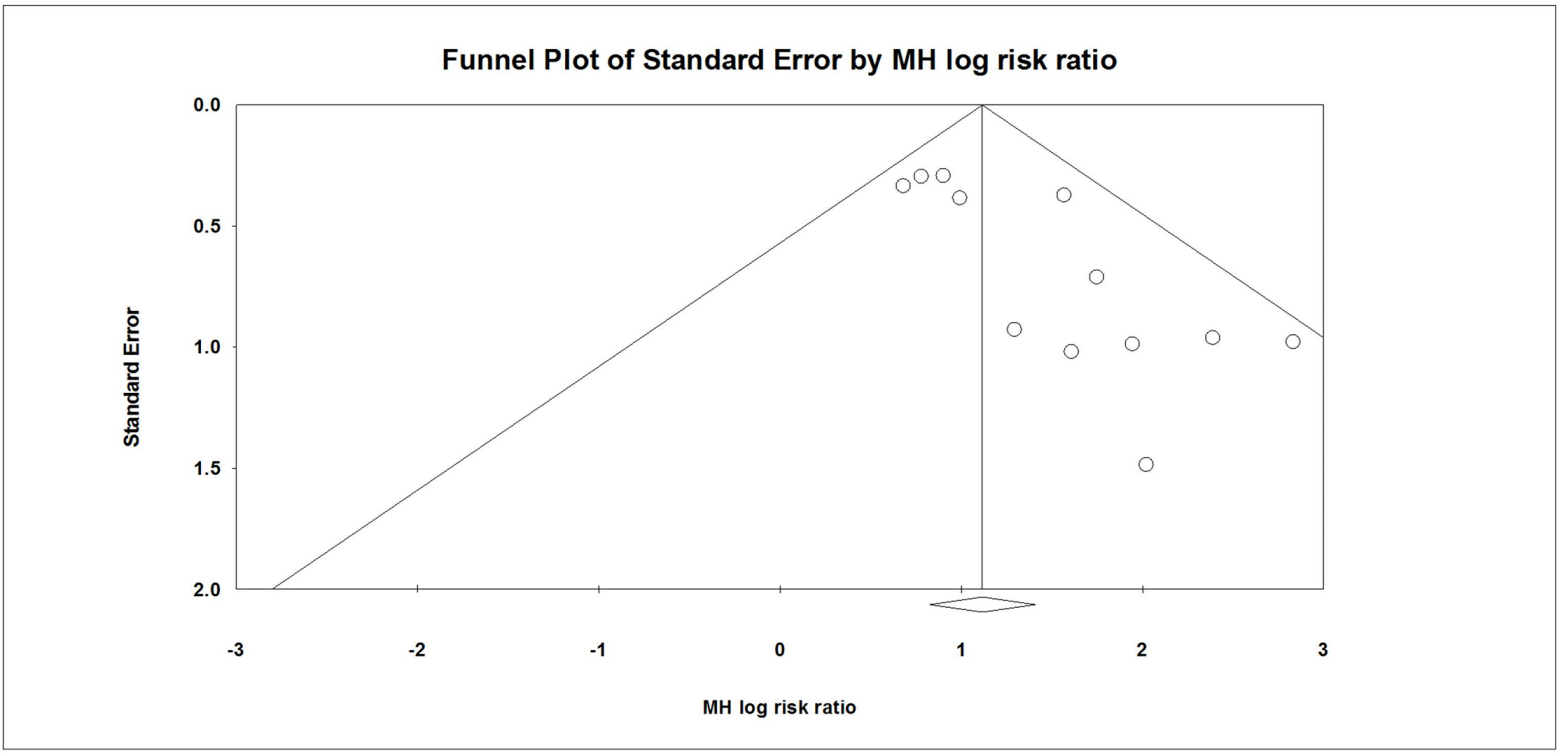
Funnel plot of studies that compared amnestic effects between benzodiazepines and placebos. Egger’s test: *P* = 0.001.

The funnel plot for these data demonstrates that the included studies were asymmetrically distributed and that there was a lack of small studies falling toward the left of the mean effect. These results are in agreement with Egger's test, which revealed there was statistically significant publication bias (*P* = 0.001) (Fig 4).

The quality of the evidence was moderate, indicating a moderate level of confidence in the effect estimate. Further research is therefore likely to both impact confidence in the estimate of the effect and change the estimate [25]. The quality of the evidence was downgraded because of concerns about publication bias (Table 2).

2. Single benzodiazepine versus single benzodiazepine

Benzodiazepines were compared to each other in 17 studies [7,18,27,29,31,39,41,43,53,54,55,57,59,61,62,64,67]. Six of these studies compared different benzodiazepines [18,39,43,53,57,62], six compared dosages [29,31,41,55,61,64], one compared the time of administration [7], and 4 compared routes [27,54,59,67]. Only four studies found significant differences among groups: in one study, intravenous midazolam was more likely than intravenous diazepam to produce amnesia for pain, but there was no difference with regard for amnesia of events [39]. Other studies have shown that anterograde amnesia is more likely to occur when oral midazolam is used than when oral diazepam was used [53], when oral flunitrazepam was used than when oral diazepam was used [62], and when a higher dose of triazolam was delivered [29].

**Fig 5:**
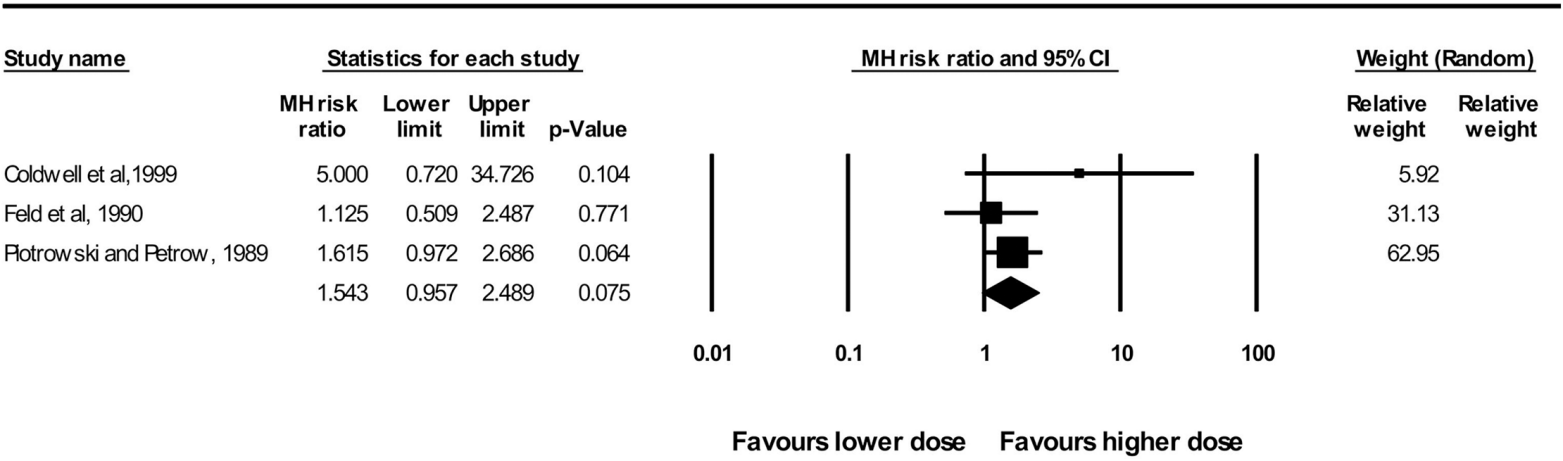
Forest plot of the meta-analysis performed to compare amnestic effects between dosages of benzodiazepines. Overall effect: RR = 1.54; 95% CI: 0.96–2.49; *P =* 0.07. Heterogeneity: I^2^ = 12%, *P* = 0.32.

A meta-analysis of 3 studies (n = 55) that compared dosages demonstrated that higher doses of benzodiazepines did not favor the occurrence of anterograde amnesia compared to lower doses (RR = 1.54; 95% CI: 0.96–2.49; *P =* 0.07; I^2^ = 12%) (Fig 5).

The quality of evidence was low, indicating that because there was limited confidence in the effect estimate, further research is very likely to both impact confidence in the estimate of the effect and change the estimate [25]. The quality of the evidence was therefore downgraded because of the degree of imprecision that was observed in these studies (Table 2).

3. Single benzodiazepine versus non-benzodiazepine drug

Benzodiazepines were compared to non-benzodiazepines in 13 studies [18,44,45,48,50,51,56–60,70,71]. In four studies, anterograde amnesia was significantly more likely when benzodiazepines were used than when nitrous oxide [50], antihistamines [45,48,60], or triclofos [48] were used. However, one study showed the anterograde amnesia was less likely to occur when benzodiazepines were used than when butorphanol was used [71].

The quality of the evidence was judged as low in consideration of the degree of imprecision that was observed (Table 2).

4. Benzodiazepine in combination with another drug versus any sedative

Benzodiazepines were used in combination with another drug and compared to any sedative in 11 studies [14–16,26,28,34,37,42,44,47,70]. Only three studies found that anterograde amnesia was significantly more likely in the benzodiazepine group than when another sedative, including meperidine, promethazine and chlorpromazine [26], meperidine alone [28], or melatonin plus nitrous oxide [42] was used. One study showed that anterograde amnesia was more likely when propofol was used than when midazolam was used [47].

A meta-analysis was not performed because there was substantial clinical heterogeneity.

Given the imprecision that was observed in these studies, the quality of evidence was determined to be low (Table 2).

5. Non-benzodiazepine drugs versus non-benzodiazepine drugs

**Fig 6:**
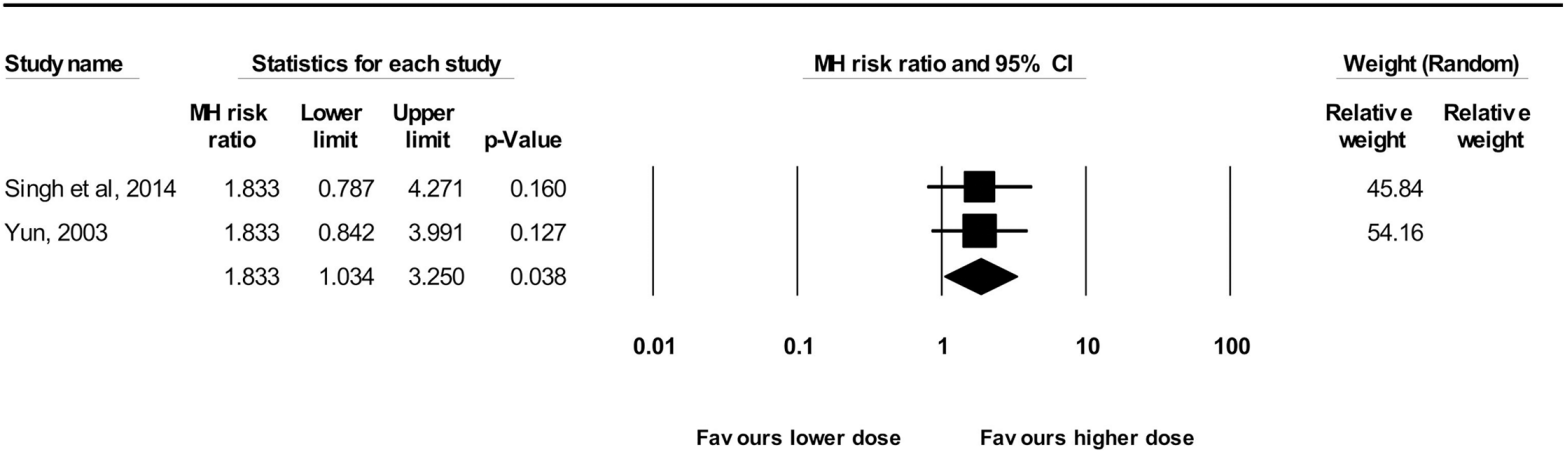
Forest plot of the meta-analysis performed to compare amnestic effects between dosages of alpha2-adrenergic agonists. Overall effect: RR = 1.83; 95% CI: 1.03–3.25; *P =* 0.04; I^2^ = 0%, *P* = 1.000.

Eight studies compared non-benzodiazepine drugs [13,17,30,38,42,46,49,65]. Anterograde amnesia was more likely when nitrous oxide/propofol was used instead of propofol/lidocaine [46], or oral ketamine was used instead of oral dexmedetomidine [49]. Considering the alpha2-adrenergic agonists (clonidine 2mcg/kg versus 1mcg/kg PO and dexmedetomidine 5mcg/kg versus 3mcg/kg PO), a meta-analysis of 2 studies (n = 96) found that higher doses of them increased occurrence of anterograde amnesia compared to lower doses (RR = 1.83; 95% CI: 1.03–3.25; *P =* 0.038; I^2^ = 0%) (Fig 6).

Imprecision downgraded the quality of the evidence to low (Table 2).

#### Retrograde amnesia

Retrograde amnesia was assessed in ten studies [7,33,35,37,39,40,52,54,60,66]. Six studies compared midazolam to placebo [7,33,35,40,52,66] and showed contradictory results: in one study, greater recall was reported after oral midazolam (0.5 mg/kg) was used [7], while the other studies found that patients had equivalent recall after midazolam was administered via different routes [33,40,52,66] or a lower rate of recall after oral midazolam (0.5 mg/kg) was used [35]. No differences were reported in other studies that compared midazolam to diazepam [39], midazolam delivered via two routes [54], benzodiazepine delivered with antihistamine [60], or ketamine delivered in different doses with diazepam [37].

The quality of the evidence in this group was low (Table 2).

## Discussion

This systematic review was performed to determine the level of scientific evidence to support the amnestic effects of different sedatives in pediatric patients. While some commonly used drugs do appear to have the potential to induce anterograde amnesia, the only group of studies that provided more than a low level of evidence were those that compared benzodiazepines to placebos, both when the sedative was used as premedication or as agent for procedural sedation. The findings related to retrograde amnesia remain controversial and with low level of evidence.

In nearly all of the included trials, memory was assessed using the study-test paradigm, which included a learning phase, a retention interval, and a test phase [5]. However, trials differed greatly in the target materials presented during learning and the duration of the retention interval. Differences between memory tasks are to be expected because there is currently no accepted standard reference memory test [5]. It can therefore be argued that a subject’s responses during a task aimed at evaluating the recall of materials, such as pictures and toys, may not reflect their responses in real life situations [5]. Nevertheless, the current systematic review found no difference in the rate of amnesia between events and pictures. Some studies have assessed the recall of both events and pictures, and there was no difference in the rate of amnesia achieved according to stimuli.

Pediatric patients that received benzodiazepines were three times more likely to experience anterograde amnesia than those who received a placebo. The likelihood of experiencing anterograde amnesia was higher when the sedatives were used as agent for procedural sedation than when used as premedication. In fact, it is widely believed that benzodiazepines impair the retention of memories of information acquired after the administration of a benzodiapine drug [72]. According to the present results, the benzodiazepine-related amnesia is independent of the method employed to evaluate it, including whether the means of recall involved pictures or events. It has been shown that memory impairments remain even when a task that mimics a real world situation is used [52,73]. Hence, benzodiazepines impair memories of study items in addition to personally relevant events [52,73].

The apparent lack of dose response of anterograde amnesia induced by benzodiazepines might be due to the fact that the lowest doses of benzodiazepines can cause sufficient amnesia, thus increasing the dose does not increase amnesia. However, the results related to dose-dependent amnesia should be viewed with skepticism due to the poor nature and imprecision of the 3 studies included in the meta-analyses. The level of evidence for this analysis was low, and it should be noted that the lower limit of the confidence interval was 0.96. Similarly, even though there was a significant dose-related amnesia in children that received alpha2-adrenergic agonists in the 2 studies included, in agreement with a previous animal study [74], the level of evidence was also low, with the lower limit of the confidence interval being 1.03.

The studies included in this systematic review provided different results regarding amnesia related to information that was acquired before sedative administration (retrograde amnesia). Interestingly, whereas nearly all of the studies reported equivalent recall when a benzodiazepine was compared with placebo or active drug [52,66,33,39,54,60,37,40], one trial demonstrated that using a sedative increased recall [7]. Improved recall of material acquired before the administration of a benzodiazepine has been reported in previous laboratory-based studies. In these cases, retrograde facilitation may be secondary to anterograde amnesia: the reduced ability to learn information after drug intake has been associated with lower levels of interference and a decrease in the chance of forgetting information that was acquired before drug intake [75,76].

Despite the importance of including a placebo control group when assessing the influence of a drug on learning and performance, nearly 70% of the included studies failed to use a placebo, probably for ethical reasons. Nonetheless, among the non-placebo-controlled studies, almost all of the trials (n = 30/36) included a group that was treated with a benzodiazepine, which is considered a standard drug for comparisons with other sedatives [12]. When considering a comparison between benzodiazepines, two studies found that greater anterograde amnesia was produced by midazolam than diazepam, and these findings were in accordance with those reported in another systematic review [77].

It is indisputable that the gold standard design for drug studies is a randomized, parallel, placebo-controlled and double-blinded trial [12]. The current systematic review considered results from only randomized controlled trials. To identify relevant studies, including both published and unpublished studies, a sensitive search strategy was implemented and used to search in different electronic databases without restrictions on language or the date of publication. Additionally, all steps were performed by two trained and calibrated reviewers, and this minimized errors and reduced potential biases [5].

The present systematic review and meta-analysis has some limitations. First, in several studies (n = 45), memory was not a primary outcome, indicating that it was not the most important outcome that was examined and that it was therefore not a basis for estimating the sample size [78]. This could have compromised the external validity of the results of these studies. Second, it was not possible to rule out the possibility that publication bias could have impaired the results of the meta-analysis. However, efforts were made to obtain unpublished, potentially relevant articles. Third, five potentially eligible papers could not be captured. However, because a large number of articles were assessed in this review, it should not be expected that the inclusion of a small number of additional articles would have altered the results. Finally, the studies included in the meta-analyses presented some clinical heterogeneity based on the age ranges of their participants and the methods used to assess amnesia. Nevertheless, these features did not impact our results according to the sensitivity analysis.

The evidence used to show that benzodiazepines produce greater rates of anterograde amnesia than placebo were of moderate quality, regardless of the age of the participants, the method used to assess memory, and the stimulus (study items or real events) being evaluated. However, the broad range of sedatives that were used in these studies and the wide variety of drug comparisons that were evaluated resulted in significant heterogeneity among the studies. Additionally, the evidence for other sedatives was of low quality and possessed limited generalizability. For these reasons, benzodiazepines can be considered a preferred option in a clinical setting when amnesia is desired.

## Conclusions

In this systematic review, we found that randomized clinical trials investigating the amnestic effects of sedatives in pediatric patients are heterogeneous, which made it difficult to obtain a high level of evidence to support conclusions relating to this topic. Nevertheless, the anterograde amnesia that is produced by benzodiazepines is well-demonstrated, and the likelihood of anterograde amnesia is higher when these sedatives are used as agents for procedural sedation than when used as premedication: the quality of evidence supporting its efficacy is moderate. The evidence for other sedatives is based only on isolated and small trials, and it should therefore be viewed with caution. The lack of high-quality evidence regarding the amnestic effects of non-benzodiazepine sedatives in children/adolescents suggests that future randomized clinical trials aimed at studying pediatric sedation should include amnesia (or, more accurately measures of memory performance) as the primary or key secondary end-point. Recommendations regarding the quality of methodology used to assess memory function should be adhered to closely.

## Supporting information

S1 Protocol Copy of the registration protocol in PROSPERO. (PDF)

S1 Appendix PRISMA checklist. (DOC)

S1 Table Search strategy used for some database searches. (DOC)

S2 Table Amnestic effects: Comparisons between benzodiazepines and placebos. (DOC)

S3 Table Amnestic effects of benzodiazepines: Comparison among benzodiazepines. (DOC)

S4 Table Amnestic effects: Comparisons between benzodiazepines and non-benzodiazepine sedatives. (DOC)

S5 Table Amnestic effects: Comparisons between benzodiazepines in combination with other drugs and any sedative. (DOC)

S6 Table Amnestic effects: Comparisons of non-benzodiazepine drugs and non-benzodiazepine drugs. (DOC)
