# Supplementary material for: What is the level of evidence for the amnestic effects of sedatives in pediatric patients? A systematic review and meta-analyses
Source: PLoS One. 2017 Jul 7;12(7):e0180248. doi: 10.1371/journal.pone.0180248 (PMC5501513; doi:10.1371/journal.pone.0180248)
Supplement: S1 Protocol — (PDF) [file pone.0180248.s001.pdf]

PROSPERO International prospective register of systematic reviews

Review title and timescale

1 Review title
Give the working title of the review. This must be in English. Ideally it should state succinctly the interventions or exposures being reviewed and the associated health or social problem being addressed in the review.
Amnestic effects of sedatives in children and adolescents undergoing procedural sedation

2 Original language title
For reviews in languages other than English, this field should be used to enter the title in the language of the review. This will be displayed together with the English language title.

3 Anticipated or actual start date
Give the date when the systematic review commenced, or is expected to commence.
01/04/2015

4 Anticipated completion date
Give the date by which the review is expected to be completed.
31/03/2016

5 Stage of review at time of this submission
Indicate the stage of progress of the review by ticking the relevant boxes. Reviews that have progressed beyond the point of completing data extraction at the time of initial registration are not eligible for inclusion in PROSPERO. This field should be updated when any amendments are made to a published record.

The review has not yet started √

Review stage	Started	Completed
Preliminary searches	Yes	Yes
Piloting of the study selection process	Yes	Yes
Formal screening of search results against eligibility criteria	Yes	Yes
Data extraction	Yes	Yes
Risk of bias (quality) assessment	Yes	Yes
Data analysis	Yes	Yes

Provide any other relevant information about the stage of the review here.
Submission on manuscript.

Review team details

6 Named contact
The named contact acts as the guarantor for the accuracy of the information presented in the register record.
Professor Costa

7 Named contact email
Enter the electronic mail address of the named contact.
lsucasas@ufg.br

8 Named contact address
Enter the full postal address for the named contact.
Faculdade de Odontologia, Primeira Avenida, sem numero, Setor Universitario Goiania-GO, Brasil CEP 74605-220

9 Named contact phone number
Enter the telephone number for the named contact, including international dialing code.
+55 62 3209-6065

10 Organisational affiliation of the review
Full title of the organisational affiliations for this review, and website address if available. This field may be completed as 'None' if the review is not affiliated to any organisation.

Universidade Federal de Goiás - UFG

Website address:

11 Review team members and their organisational affiliations

Give the title, first name and last name of all members of the team working directly on the review. Give the organisational affiliations of each member of the review team.

Title	First name	Last name	Affiliation
Professor	Luciane	Costa	Universidade Federal de Goiás - UFG
Dr	Karolline	Viana	Universidade Federal de Goiás - UFG
Dr	Anelise	Daher	Universidade Federal de Goiás - UFG
Professor	Lucianne	Maia	Universidade Federal do Rio de Janeiro - UFRJ
Professor	Paulo	Costa	Universidade Federal de Goiás - UFG
Professor	Carolina	Martins	Universidade Federal de Minas Gerais - UFMG
Professor	Saul	Paiva	Universidade Federal de Minas Gerias - UFMG

12 Funding sources/sponsors

Give details of the individuals, organizations, groups or other legal entities who take responsibility for initiating, managing, sponsoring and/or financing the review. Any unique identification numbers assigned to the review by the individuals or bodies listed should be included.

Fundação de Amparo à Pesquisa do Estado de Goiás (FAPEG) Coordenação de Aperfeiçoamento de Pessoal de Nível Superior (CAPES) Conselho Nacional de Desenvolvimento Científico e Tecnológico (CNPq)

13 Conflicts of interest

List any conditions that could lead to actual or perceived undue influence on judgements concerning the main topic investigated in the review.

Are there any actual or potential conflicts of interest?

None known

14 Collaborators

Give the name, affiliation and role of any individuals or organisations who are working on the review but who are not listed as review team members.

Title	First name	Last name	Organisation details

Review methods

15 Review question(s)

State the question(s) to be addressed / review objectives. Please complete a separate box for each question.

What is the effect of different sedatives on memory of intraoperative events in children and adolescents undergoing procedural sedation?

16 Searches

Give details of the sources to be searched, and any restrictions (e.g. language or publication period). The full search strategy is not required, but may be supplied as a link or attachment.

Two reviewers will independently perform the search process under the guidance of a librarian. The following electronic databases will be searched: PubMed, Scopus, The Cochrane Library, Web of Science, LILACS. MeSh terms, key words and other free terms related to memory and sedation will be used with Boolean operators (OR, AND) to combine searches. There will be no language restrictions. Reference lists of all included studies will be hand searched in order to locate additional studies. Articles appearing in more than one database search will be considered only once.

17 URL to search strategy

If you have one, give the link to your search strategy here. Alternatively you can e-mail this to PROSPERO and we will store and link to it.

I give permission for this file to be made publicly available
Yes

18 Condition or domain being studied
Give a short description of the disease, condition or healthcare domain being studied. This could include health and wellbeing outcomes.
This question is important because many persons remain conscious during a procedural sedation and, sometimes, they can show uncooperative behaviour and/or anxiety although sedated. So, if a sedated child keeps crying and/or moving during the procedure, will s/he remember the interventions performed? Will s/he recall any aversive stimuli received during the procedure and then be traumatized by it? If a sedated person does not remember any intraoperative event, this is good news for patients/parents and clinicians, because it confirms the benefit of amnesia associated with procedural conscious sedation, although there is a chance that the sedative does not keep patients quiet or calm. The relevance of this question is that if we have evidence for amnesia related to paediatric conscious sedation, we are assured that if children and adolescents show distress reactions during procedural sedation, they will not register it as a negative experience, and probably will have less chance of a future psychological trauma.

19 Participants/population
Give summary criteria for the participants or populations being studied by the review. The preferred format includes details of both inclusion and exclusion criteria.
Children and adolescents: - aged 2 to 19 years old - without cognitive or impairment - receiving sedatives as premedication or as agents for conscious procedural sedation in a medical or dental setting

20 Intervention(s), exposure(s)
Give full and clear descriptions of the nature of the interventions or the exposures to be reviewed
Any sedative regimen administered by a health professional in an outpatient setting, dental office or operating room

21 Comparator(s)/control
Where relevant, give details of the alternatives against which the main subject/topic of the review will be compared (e.g. another intervention or a non-exposed control group).
Placebo Variations of the same sedative regimen (dosage, route and timing of administration) Alternative sedation regimen

22 Types of study to be included
Give details of the study designs to be included in the review. If there are no restrictions on the types of study design eligible for inclusion, this should be stated.
Randomized clinical trials

23 Context
Give summary details of the setting and other relevant characteristics which help define the inclusion or exclusion criteria.

24 Primary outcome(s)
Give the most important outcomes.
Anteretrograde amnesia, defined as loss of memory of the procedure assessed through recall and recognition tests performed after sedative administration

Give information on timing and effect measures, as appropriate.

25 Secondary outcomes
List any additional outcomes that will be addressed. If there are no secondary outcomes enter None.
Retrograde amnesia

Give information on timing and effect measures, as appropriate.

26 Data extraction (selection and coding)
Give the procedure for selecting studies for the review and extracting data, including the number of researchers involved and how discrepancies will be resolved. List the data to be extracted.

27 Risk of bias (quality) assessment
State whether and how risk of bias will be assessed, how the quality of individual studies will be assessed, and

whether and how this will influence the planned synthesis.
Five possible sources of bias in the conduct and reporting of controlled trials are selection bias, performance bias, detection bias, attrition bias and reporting bias. We will attempt to keep the effect of these biases to a minimum by having at least two persons extracting data independently. They will use the ‘Risk of bias’ tool (Cochrane).

28 Strategy for data synthesis
Give the planned general approach to be used, for example whether the data to be used will be aggregate or at the level of individual participants, and whether a quantitative or narrative (descriptive) synthesis is planned. Where appropriate a brief outline of analytic approach should be given.
The appropriateness of running a meta-analysis will be checked. Data synthesis will be summarized in tables stratified by children's age range.

29 Analysis of subgroups or subsets
Give any planned exploration of subgroups or subsets within the review. ‘None planned’ is a valid response if no subgroup analyses are planned.
If there are sufficient data, we plan to conduct subgroup analysis for age, considering the different cognitive abilities among age ranges (Piaget): 2 to under 7 years old, 7 to under 12 years old, 12 to 19 years old.

Review general information

30 Type and method of review
Select the type of review and the review method from the drop down list.
Intervention, Systematic review

31 Language
Select the language(s) in which the review is being written and will be made available, from the drop down list. Use the control key to select more than one language.
English

Will a summary/abstract be made available in English?
Yes

32 Country
Select the country in which the review is being carried out from the drop down list. For multi-national collaborations select all the countries involved. Use the control key to select more than one country.
Brazil

33 Other registration details
Give the name of any organisation where the systematic review title or protocol is registered together with any unique identification number assigned. If extracted data will be stored and made available through a repository such as the Systematic Review Data Repository (SRDR), details and a link should be included here.

34 Reference and/or URL for published protocol
Give the citation for the published protocol, if there is one.
Viana KA, Daher A, Maia LC, Costa PS, Martins CC, Paiva SM, Costa LR. Memory effects of sedative drugs in children and adolescents--protocol for a systematic review. Syst Rev. 2016 Feb 18;5:34. doi: 10.1186/s13643-016-0192-x.

Give the link to the published protocol, if there is one. This may be to an external site or to a protocol deposited with CRD in pdf format.
https://systematicreviewsjournal.biomedcentral.com/articles/10.1186/s13643-016-0192-x

I give permission for this file to be made publicly available
Yes

35 Dissemination plans
Give brief details of plans for communicating essential messages from the review to the appropriate audiences.
Do you intend to publish the review on completion?
Yes

36 Keywords
Give words or phrases that best describe the review. (One word per box, create a new box for each term)

37 Details of any existing review of the same topic by the same authors
Give details of earlier versions of the systematic review if an update of an existing review is being registered, including full bibliographic reference if possible.

38 Current review status
Review status should be updated when the review is completed and when it is published.
Completed but not published

31/07/2017

39 Any additional information
Provide any further information the review team consider relevant to the registration of the review.

40 Details of final report/publication(s)
This field should be left empty until details of the completed review are available.
Give the full citation for the final report or publication of the systematic review.
Give the URL where available.
