# Supplementary material for: What is the level of evidence for the amnestic effects of sedatives in pediatric patients? A systematic review and meta-analyses
Source: PLoS One. 2017 Jul 7;12(7):e0180248. doi: 10.1371/journal.pone.0180248 (PMC5501513; doi:10.1371/journal.pone.0180248)
Supplement: S1 Table — (DOC) [file pone.0180248.s003.doc]

Search strategy used for some database searches

| Database | Search Strategy |
| --- | --- |
| PubMed  (http://www.ncbi.nlm.nih.gov/pubmed) | Available in the previous published protocol.^20^ |
| Web of Science  (http://webofknowledge.com/) | # 6 #5 AND #4 AND #1  # 5 TOPIC: ("Amnesia anterograde") OR TOPIC: (Amnesia) OR TOPIC: ("States Amnestic") OR TOPIC: ("Memory Loss") OR TOPIC: ("Amnesia retrograde") OR TOPIC: ("Memory Episodic") ORTOPIC: ("Memory Autobiographical") OR TOPIC: ("Prospective Memory") OR TOPIC: (Memory) OR TOPIC: ("Memory short-term") OR TOPIC: ("Short-Term Memory") OR TOPIC: ("Working Memory") OR TOPIC: ("Sensory Memory") OR TOPIC: ("Memory long-term") OR TOPIC: ("Explicit Memory") OR TOPIC: ("Declarative Memory") OR TOPIC: ("Semantic Memory") OR TOPIC:("Nondeclarative Memory") OR TOPIC: ("Procedural Memory") OR TOPIC: (Recall) OR TOPIC: ("Repetition priming") OR TOPIC: ("Priming Repetition") OR TOPIC: ("Implicit memory")  # 4 #3 OR #2  # 3 TOPIC: ("Anesthetics Dissociative") OR TOPIC: (Dexmedetomidine) OR TOPIC: (Meperidine) OR TOPIC: (Pethidine) OR TOPIC: (Fentanyl) OR TOPIC: ("Adjuvants Anesthe*") OR TOPIC:(Morphine) OR TOPIC: ("Morphine Derivatives") OR TOPIC: (Ketamine) OR TOPIC: ("Ketorolac Tromethamine") OR TOPIC: (Ketorolac) OR TOPIC: (Indomethacin) OR TOPIC: (Tolmetin) ORTOPIC: ("Chloral hydrate") OR TOPIC: (Hydroxyzine) OR TOPIC: (Promethazine) OR TOPIC: (Phenergan) OR TOPIC: (Propofol) OR TOPIC: ("Anesthetics inhalation") OR TOPIC: ("Gases Anesthetic") OR TOPIC: (Sevoflurane)  # 2 TOPIC: (Hypnotic*) OR TOPIC: (Sedative*) OR TOPIC: (Tranquilizer) OR TOPIC: ("Histamine H1 Antagonists") OR TOPIC: ("Antagonists Histamine H1") OR TOPIC: ("Antihistamines Classical") ORTOPIC: ("Antihistamines Sedating") OR TOPIC: (Tranquilizing) OR TOPIC: (Narcotic*) OR TOPIC: (Anesthetic*) OR TOPIC: (Anaesthetic*) OR TOPIC: (Opioid*) OR TOPIC: ("Tranquillizing Agents Minor") OR TOPIC: ("Anti-Anxiety") OR TOPIC: (Antianxiety) OR TOPIC: (Anxiolytic*) OR TOPIC: (Benzodiazepine*) OR TOPIC: (Midazolam) OR TOPIC: (Diazepam) OR TOPIC: ("Nitrous oxide")OR TOPIC: (N2O) OR TOPIC: ("Relative analgesia") OR TOPIC: (Barbiturates) OR TOPIC: ("Anesthetics Intravenous") OR TOPIC: ("Intravenous Anesthetics")  # 1 Tópico: (Child*) OR Tópico: (Pediatric) OR Tópico: (Paediatric) OR Tópico: ("Child preschool") OR Tópico: ("Preschool Child*") OR Tópico: (Adolescen*) OR Tópico: (Youth*) |
| Scopus  (http://www.scopus.com/) | ( ( TITLE-ABS-KEY ( child*) OR TITLE-ABS-KEY ( pediatric) OR TITLE-ABS-KEY ( paediatric) OR TITLE-ABS-KEY ( "Chid preschool") OR TITLE-ABS-KEY ( "Preschool Child*") OR TITLE-ABS-KEY ( adolescen*) OR TITLE-ABS-KEY ( youth* ) ) ) AND ( ( TITLE-ABS-KEY ( hypnotic*) OR TITLE-ABS-KEY ( sedative*) OR TITLE-ABS-KEY ( tranquilizer) OR TITLE-ABS-KEY ( " Histamine H1 Antagonists") OR TITLE-ABS-KEY ( " Antagonists Histamine H1") OR TITLE-ABS-KEY ( " Antihistamines Classical") OR TITLE-ABS-KEY ( " Antihistamines Sedating") OR TITLE-ABS-KEY ( tranquilizing) OR TITLE-ABS-KEY ( narcotic*) OR TITLE-ABS-KEY ( anesthetic*) OR TITLE-ABS-KEY ( anaesthetic*) OR TITLE-ABS-KEY ( opioid*) OR TITLE-ABS-KEY ( " Tranquillizing Agents") OR TITLE-ABS-KEY ( " Anti-Anxiety") OR TITLE-ABS-KEY ( " Antianxiety") OR TITLE-ABS-KEY ( anxiolytic*) OR TITLE-ABS-KEY ( benzodiazepine*) OR TITLE-ABS-KEY ( midazolam) OR TITLE-ABS-KEY ( diazepam) OR TITLE-ABS-KEY ( " Nitrous oxide") OR TITLE-ABS-KEY ( "N2O") OR TITLE-ABS-KEY ( "Relative analgesia") OR TITLE-ABS-KEY ( barbiturates) OR TITLE-ABS-KEY ( " Anesthetics Intravenous") OR TITLE-ABS-KEY ( "Intravenous Anesthetics") OR TITLE-ABS-KEY ( " Anesthetics Dissociative") OR TITLE-ABS-KEY ( dexmedetomidine) OR TITLE-ABS-KEY ( meperidine) OR TITLE-ABS-KEY ( pethidine) OR TITLE-ABS-KEY ( fentanyl) OR TITLE-ABS-KEY ( "Adjuvants Anesthe*") OR TITLE-ABS-KEY ( morphine) OR TITLE-ABS-KEY ( "Morphine Derivatives") OR TITLE-ABS-KEY ( ketamine) OR TITLE-ABS-KEY ( "Ketorolac Tromethamine") OR TITLE-ABS-KEY ( ketorolac) OR TITLE-ABS-KEY ( indomethacin) OR TITLE-ABS-KEY ( tolmetin) OR TITLE-ABS-KEY ( "Chloral hydrate") OR TITLE-ABS-KEY ( hydroxyzine) OR TITLE-ABS-KEY ( promethazine) OR TITLE-ABS-KEY ( phenergan) OR TITLE-ABS-KEY ( propofol ) OR TITLE-ABS-KEY ( "Anesthetics inhalation") OR TITLE-ABS-KEY ( "Gases Anesthetic") OR TITLE-ABS-KEY ( sevoflurane ) ) ) AND ( ( TITLE-ABS-KEY ( "amnesia anterograde") OR TITLE-ABS-KEY ( " Memory Loss Anterograde") OR TITLE-ABS-KEY ( "amnesia") OR TITLE-ABS-KEY ( "states amnestic") OR TITLE-ABS-KEY ( "amnesia retrograde") OR TITLE-ABS-KEY ( "memory episodic") OR TITLE-ABS-KEY ( "Memory Autobiographical") OR TITLE-ABS-KEY ( "prospective memory") OR TITLE-ABS-KEY ( memory) OR TITLE-ABS-KEY ( "Memory short-term") OR TITLE-ABS-KEY ( "Short-Term Memory") OR TITLE-ABS-KEY ( "Working Memory") OR TITLE-ABS-KEY ( "Sensory Memory") OR TITLE-ABS-KEY ( "Memory long-term") OR TITLE-ABS-KEY ( " Explicit Memory") OR TITLE-ABS-KEY ( "Declarative Memory") OR TITLE-ABS-KEY ( "Semantic Memory") OR TITLE-ABS-KEY ( "Nondeclarative Memory") OR TITLE-ABS-KEY ( "Procedural Memory") OR TITLE-ABS-KEY ( recall) OR TITLE-ABS-KEY ( "Repetition priming") OR TITLE-ABS-KEY ( "Priming Repetition") OR TITLE-ABS-KEY ( "implicit memory" ) ) ) |
