# Supplementary material for: What is the level of evidence for the amnestic effects of sedatives in pediatric patients? A systematic review and meta-analyses
Source: PLoS One. 2017 Jul 7;12(7):e0180248. doi: 10.1371/journal.pone.0180248 (PMC5501513; doi:10.1371/journal.pone.0180248)
Supplement: S2 Table — (DOC) [file pone.0180248.s004.doc]

Amnestic effects: comparisons between benzodiazepines and placebos

| **Author, year, country** | **Study design** | **Participants**  **n (age)** | **Sedative use, procedure and setting** | **Sedative regimen** | | **Type of amnesia** | **Outcome measure** | | **Outcome result/conclusion** |
| --- | --- | --- | --- | --- | --- | --- | --- | --- | --- |
|  |  |  |  | **Intervention** | **Comparison** |  |  |  |  |
| Kaviani et al, 2014, Iran [68] | Triple-blind, parallel | 62 (4-10 years old) | Premedication  Dental (not specified)  Operating room | A: midazolam (0.5 mg/kg) PO (n=30) | B: placebo (n=32) | Anterograde amnesia | | Recall of events, before discharge | - Recall of pre-anesthesia events: A: 10% (3/30); B: 81% (26/32) (*P*< 0.001).  -Recall of placement of anesthesia mask: lower in group A (*P*< 0.001).  - Recall of other operating procedures (e.g., hand contact, hearing and seeing): no difference (*P*= 0.062). |
| Hedén et al, 2009, Sweden [19] | Triple-blind, parallel | 50 (1–18 years old) | Procedural sedation  Medical (needle procedures)  Outpatient | A: midazolam (0.3 mg/kg) PO (n=24) | B: placebo (n=26) | Anterograde amnesia | | Patient-reported side effects | Amnesia: A: 12% (3/24); B: 0% (0/26) |
| Millar et al, 2007, UK [69] | Not available, parallel | 179 (5–10 years old) | Premedication  Dental (extractions)  Operating room | A: midazolam (0.2 mg/kg) MB (n=90) | B: placebo (n=89) | Anterograde amnesia | | Recall of pictures postoperatively and at 48 h. Recognition at 48 h. | Recall: Group B recalled more pictures postoperatively (*P*=0.014) and at 48 h (*P*=0.012).  Recognition: Group B recognized more stimuli (*P* < 0.001).  Amnesia postoperatively:  A: 37% (22/59); B: 17% (14/82);  Amnesia at 48h: A: 50% (28/56); B: 14% (11/79). |
| Stewart et al, 2006, Canada [63] | Double-blind, parallel | 23 (3–6 years old) | Premedication  Medical (surgery)  Operating room | A: midazolam (0.5 mg/kg) PO + acetaminophen (15 mg/kg) (n=12) | B: acetaminophen (15 mg/kg) PO (n=11) | Anterograde amnesia | | Recall of pictures, after the child was awake | Implicit memory: no main effect of the drug group was associated with priming (*P*>0.05).  Explicit memory : Group A showed poorer recognition than Group B (*P*<0.05) |

| Buffett-Jerrott et al, 2003, Canada [52] | Double-blind, parallel | 40 (4–6 years old) | Premedication  Medical (surgery)  Operating room | A: midazolam (0.5 mg/kg) + acetaminophen (15 mg/kg) PO (n=20) | B: acetaminophen (15 mg/kg) PO (n=20) | Anterograde and retrograde amnesia | Recall of pictures (±25 min and ±130 min post-drug) and events (±130 min post-drug). | - Anterograde amnesia: Group B remembered more pictures and post-drug events than Group A (*P*<0.05).  - Retrograde amnesia: no difference in recall of pre-drug events. |
| --- | --- | --- | --- | --- | --- | --- | --- | --- |
| Kain et al, 2000, USA [7] | Double-blind, parallel | 113 (5-10 years old) | Premedication  Medical (surgery)  Operating room | A: midazolam (0.5 mg/kg) + acetaminophen (10 mg/kg) PO - pictures showed at 5 min post-drug (n=25) | Acetaminophen (10 mg/kg) PO plus:  B: midazolam (0.5 mg/kg) - pictures showed 10 min post-drug (n=30).  C: midazolam (0.5 mg/kg) - 20 min post-drug (n=25) D: none - 15 min post-drug (n=33). | Anterograde and retrograde amnesia | Recall/recognition of pictures showed pre and post sedative administration and recall of events, before discharge. | -Anterograde amnesia: Recall of pictures: A: median 25.0 (25-75%; 0–33); B: 8 (0–29); C: 0 (0–0); D: 25.0 (8–33) (*P*≤0.004 for B-D; C-D).  Recognition of pictures: A: 83.3 (67–96); B: 70.8 (42–92); C: 41.6 (4–75); D: 91.6 (91.6–100) (*P*≤ 0.0008 for A-D; B-D; C-D).  Recall of pulse oximeter: A: 39%; B: 36%; C: 17%; D: 72% (*P≤*0.02 for A-D; B-D; C-D).  Recall of the electrocardiography leads: A: 41%; B: 26%; C: 27%; D 61% (*P*≤0.015 for B-D; C-D).  - Retrograde amnesia: Recall of pictures: A: 12.5 (8–33); B: 25 (8–42); C: 33 (16–50): D: 12.5 (0–25) (*P*≤0.002 for B-D; C-D). Recognition: no difference |
| Raadal et al, 1999, USA [36] | Double-blind, parallel | 54 (3 -5 years old) | Procedural sedation  Dental (restorations and/or extractions )  Outpatient | A: triazolam (0.03 mg/kg) PO (n=27) | B: placebo (n=27) | Anterograde amnesia | Recall of a toy, at 90 min post-drug administration. | Amnesia: 50% (10/ 20); B: 9% (2/ 23) (*P* = 0.003). |

| Liacouras et al, 1998, USA [35] | Double-blind, parallel | 123 (≥1 years old) | Premedication  Medical (endoscopy procedures)  Outpatient | A: midazolam (0.5 mg/kg) PO (n=61) | B: placebo (n=62) | Anterograde and retrograde amnesia | Recall of objects, before discharge and on the next day (children ≥ 5 years). | Anterograde amnesia: A: 91% (32/35); B: 87%(33/38) (*P*=0.546)  Retrograde amnesia: A: 83% (29/35); B: 37% (14/38) (*P*= 0.001). |
| --- | --- | --- | --- | --- | --- | --- | --- | --- |
| Twersky et al, 1993, USA [40] | Double-blind, parallel | 34 (4-6 years old) | Premedication  Medical (surgery)  Operating room | A: midazolam (0.2 mg/kg) IN (n=15) | B: placebo (n=19) | Anterograde and retrograde amnesia | Recall/recognition of pictures after two h. | Anterograde amnesia: Recall: A: median 1.78; B: 4.01 (P < .003). Recognition: A: 6.64; B: 14.39 (*P <*0.001)  Retrograde amnesia: Recall: A: 5.84; B: 6.8 (*P*>0.05); Recognition: A: 15.22; B: 15.29 (*P*>0.05) |
| Dubost et al, 1991, France [66] | Double-blind, parallel | 40 (4-10 years old) | Premedication  Dental (maxillofacial surgery)  Operating room | A: Atropine (0.015 mg/kg) + midazolam (0.3-0.5 mg/kg) IM (n=20) | B: Atropine 0.015 mg/kg+injectable water (0.3-0.5 mg/kg) IM (n=20) | Anterograde and retrograde amnesia | Recall of a drawing and a photograph, at 6 and at 24 h. | Anterograde amnesia: A: 85% (17/20); B: 5% (1/20) (*P*<0.001)  Retrograde amnesia: A: 5% (1/20); B: 0/20 (*P*>0.05). |
| Friedman et al, 1991, USA [33] | Double-blind, parallel | 23 (3-16 years old) | Procedural sedation  Medical (aspiration and puncture)  Outpatient | A: midazolam (0.2 mg/kg) IV (n=11) | B: placebo IV (n=12) | Anterograde and retrograde amnesia | Recall of cards after one h. | Anterograde amnesia: no recall: A: 88%; B: 9% (*P*<0.001); no recognition: A: 100%; B: 18% (*P*<0.001).  Retrograde amnesia: equivalent recall and recognition (*P*>0.25). |
| Payne et al, 1991, South Africa [59] | Not available, parallel | 128 (3-10 years old) | Premedication  Medical (surgery)  Operating room | A: midazolam (0.15 mg/kg) IM (n=33) | B: no premedication (n=31); C: trimeprazine (2 mg/kg) + methadone (0.1 mg/kg) +droperidol (0.15 mg/kg) PO (n=32); D: midazolam (0.45 mg/kg) PO (n=32) | Anterograde amnesia | Recall of pictures and events after 24 h | Amnesia for pictures: A: 61% (20/33); B: 16% (5/31); C: 44 % (14/32); D: 59% (19/32) (*P*<0.001 for B-D and A-B; *P*<0.05 for C-B).  Recall of induction: A: 45% (15/33); B: 81% (25/31); C: 66% (21/32); D: 50% (16/32) (*P*<0.05 for A-B and B-D; *P*>0.05 to C-B). |

| Feld et al, 1990, USA [31] | Double-blind, parallel | 124 (1-10 years old) | | Premedication  Medical (surgery)  Operating room | | A: Atropine (0.03 mg/kg) + midazolam (0.25 mg/kg) PO (n=31) | | Atropine 0.03 mg/kg plus:  B: midazolam vehicle PO (n=31); C: midazolam (0.5 mg/kg) PO (n=31); D: midazolam (0.75 mg/kg) PO (n=31) | | Anterograde amnesia | | Recall of picture by children ≥5 years, prior to discharge. Questionnaire about events given on the next day. | | Recall the picture: A: 75%; B: 76%; C: 88%; D: 30% (*P*<0.05 for D-A; D-B; D-C)  Recall of "going to sleep": A: 17%; B: 67%; C: 33%; D: 22% (*P*<0.05 for A-B and D-B).  Recall of the face mask application: A: 40%; B: 83%; C: 40%; D: 36% (*P*< 0.05 for D-B). | |
| --- | --- | --- | --- | --- | --- | --- | --- | --- | --- | --- | --- | --- | --- | --- | --- |
| Van de Velde et al, 1987, Belgium [64] | Open label, parallel | 30 (5-10 years old) | | Premedication  Medical (surgery)  Operating room | | A: lorazepam 1 mg given by FDDF (fast dissolving dosage form) (n=10) | | B: placebo given by FDDF (n=10) C: lorazepam 2 mg given by FDDF (n=10) | | Anterograde amnesia | | Recall of picture and events, before leaving the hospital. | | Amnesia concerning venipuncture: A: 50% (5/10); B: 0% (0/10); C: 40% (4/10) (*P*<0.05 for A-B and C-B).  Amnesia concerning awakening: A: 90% (9/10); B: 30% (3/10); C: 90% (9/10) (*P*<0.05 for A-B and C-B).  Recall of receiving premedication, return to the ward, and picture: no significant difference | |
| Flaitz et al, 1986, USA [32] | Double-blind, crossover | 12 (2-6 years old) | | Procedural sedation  Dental (restorative)  Outpatient | | A: diazepam (0.6 mg/kg) PO (n=12) | | B: placebo (n=12) | | Anterograde amnesia | | Recall of a toy, ±60 min post-drug administration | | Amnesia: A: 58% (7/12); B: 8% (1/12) (*P*≤0.005). | |
| Padfield et al, 1986, UK [58] | Single-blind, parallel | | 85 (3-9 years old) | | Premedication  Medical (surgery)  Operating room | | A: trimeprazine (4 mg/kg) PO (n=29) | | B: temazepam (1 mg/kg) PO (n=29)  C: Placebo (n=27) | | Anterograde amnesia | | Recall of picture on the following day. | | Amnesia: A: 61% (17/28); B: 59% (17/29); C: 30% (8/27) (*P*=0.04 for A-C; *P*=0.06 for B-C). |
| Burtles and Astley, 1983, UK [18] | Double-blind, parallel | | 100 (5-13years old) | | Premedication  Medical (surgery)  Operating room | | A: lorazepam  (0.05 mg/kg) PO (n=25) | | B: placebo (n=25); C: trimeprazine (2.5 mg/kg) PO (n=25); D: diazepam (0.25 mg/kg) PO (n=25) | | Anterograde amnesia | | Recall of picture on the following day | | Amnesia: A: 28% (7/25); B: 4% (1/25); C: 0% (0/25); D: 12% (3/25). (*P*<0.01 for A-B; *P*>0.05 for A-D; B-D and C-D). |
| PO = oral route; MB = buccal route; IN = intranasal route; IM= intramuscular route; IV = intravenous route | | | | | | | | | | | | | | | |
