# Supplementary material for: What is the level of evidence for the amnestic effects of sedatives in pediatric patients? A systematic review and meta-analyses
Source: PLoS One. 2017 Jul 7;12(7):e0180248. doi: 10.1371/journal.pone.0180248 (PMC5501513; doi:10.1371/journal.pone.0180248)
Supplement: S3 Table — (DOC) [file pone.0180248.s005.doc]

# Amnestic effects of benzodiazepines: comparison among benzodiazepines

| **Author, year, country** | **Study design** | **Participants**  **n (age)** | **Sedative use, procedure and setting** | **Sedative regimen** | | **Type of amnesia** | **Outcome measure** | **Outcome result/conclusion** |
| --- | --- | --- | --- | --- | --- | --- | --- | --- |
|  |  |  |  | **Intervention** | **Comparison** |  |  |  |
| Aydintug et al, 2004, USA [27] | Open label, parallel | 50 (2-9 years old) | Procedural sedation  Dental (extraction)  Outpatient | A: midazolam (0.5 mg/kg) PO (n=25) | B: midazolam (0.35 mg/kg) RR (n=25) | Anterograde amnesia | Recall of pictures | Amnesia: A: 60% (15/25); B: 56% (14/25) ( *P*>0.05) |
| Jensen and Matsson, 2002, Sweden [67] | Open label, parallel | 50 (2-7 years old) | Premedication  Dental (not specified)  Operating room | A:  midazolam (0.3 mg/kg) RR (n=25) | B: midazolam (0.4 mg/kg) PO (n=25) | Anterograde amnesia | Recall of events (at 24h and 7 days) and an animal toy (at 7 days). | Recall of events: At 24 h:  A: 56% (14/25); B: 68% (17/25); At 7 days: A: 64% (16/25); B: 84% (21/25) (*P*>0.05)  Recall of toy: A: 24% (6/25); B: 40% (10/25) (*P*>0.05) |
| Coldwell et al, 1999, USA [29] | Double-blind, parallel | 30 (39–81 months old) | Procedural sedation  Dental (restorative and extraction)  Outpatient | A: triazolam (0.005 mg/kg) PO (n=10) | B: triazolam (0.015 mg/kg) PO (n=10)  C: triazolam (0.03 mg/kg) PO (n=10) | Anterograde amnesia | Recall of a toy following a 60-min delay | Amnesia: A: 11% (1/9); B: 33% (3/9); C: 56% (5/9) (*P*<0.05 - comparison of all groups). |
| Jensen et al, 1999, Sweden [43] | Double-blind, parallel | 90 (1.5-3.5 years old) | Procedural sedation  Dental (extraction)  Outpatient | A: diazepam (0.7 mg/kg) RR (n=45) | B: midazolam (0.3 mg/kg) RR (n=45) | Anterograde amnesia | Recall of events and a toy animal on the following day | Amnesia concerning the toy animal (*P*>0.05): no recall: A: 93% (38/41); B: 82% (31/38) (*P*>0.05); no recognition: A: 63% (26/41); B: 56% (22/39)  Amnesia concerning the treatment: A: 85% (28/33); B: 74% (23/31) |
| Davies and Waters, 1998, UK [41] | Open-label, parallel | 50 (1-13 years old) | Procedural sedation  Medical and dental (minor procedure)  Outpatient | A: midazolam (0.5 mg/kg) PO (n=41) | B: midazolam (0.2 mg/kg) PO (n=9) | Anterograde amnesia | Questionnaire about events, responded by parents after 2-7 days. | Amnesia: no difference between the groups. Altogether, 66% (27/42) of children had lost some memory of their attendance. |

| Chavarry Broncales et al, 1995, Peru [53] | Double-blind, parallel | 62 (1-12 years old) | Premedication  Medical (surgery)  Operating room | A: midazolam (0.2 mg/kg) PO + atropine (0.03 mg/kg) (n=31) | B: diazepam (0.2 mg/kg) PO + atropine (0.03 mg/kg) (n=31) | Anterograde amnesia | Recall of pictures by children older than 5 years old, before discharge. | Amnesia: A: 90% (28/31); B: 60% (18/31) (*P*<0.01) |
| --- | --- | --- | --- | --- | --- | --- | --- | --- |
| Tolia et al, 1990, USA [39] | Double-blind, parallel | 39 (6-18 years old) | Procedural sedation  Medical ( endoscopic)  Outpatient | A: midazolam (0.1-0.15 mg/kg) IV (n=20) | B: diazepam (0.2-0.4 mg/kg) IV (n=21) | Anterograde and retrograde amnesia | Questionnaire about recall of events and pain, applied at 1 and 24 h after the procedure | Complete amnesia: At 1 h: A: 44% (8/18); B: 71% (15/21) (*P*=0.08); At 24 h: A: 61% (11/18); B: 81 % (17/21) (*P*=0.13)  Recall of pain: Group A reported less recall of pain/discomfort at 1 (*P*=0.01) and 24 h (*P*=0.006) than Group B. |
| Piotrowski and Petrow, 1989, Switzerland [61] | Double-blind, parallel | 80 (2-10 years old) | Premedication  Medical (surgery)  Operating room | A: midazolam (0.4 mg/kg) RR + atropine (0.015-0.02 mg/kg) (n=40) | B: midazolam (0.5 mg/kg) RR +atropine (0.015-0.02 mg/kg) (n=40) | Anterograde amnesia | Recall of pictures and events by children 6-10 years old, on the night following the surgery and on the next day | Amnesia: A: 60% (6/10); B: 100 % (10/10) |
| De Jong and Verbug, 1988, The Netherlands[54] | Open-label, parallel | 202 (10 months-9 years old) | Premedication  Medical (surgery)  Operating room | A: midazolam (0.5 mg/kg) RR + atropine (0.05 mg/kg) (n=102) | B: midazolam (0.15 mg/kg) IM + atropine (0.02 mg/kg) (n=100) | Anterograde and retrograde amnesia | Recall of events pre- and post-sedative administration, at 24 h (children >3 years old). | Anterograde amnesia (*P*>0.05): operating room and induction: A: 71% (22/31); B: 50% (16/32); time spent in the recovery room: A: 74% (23/31); B: 59% (19/32)  Retrograde amnesia: A: 13% (4/31); B: 3% (1/32) (*P*>0.05) |
| Fell et al, 1985, Canada [55] | Blind, parallel | 101 children (2-12 years old) | Premedication  Medical (surgery)  Operating room | A: diazepam (0.25 mg/kg) PO (n=53) | B: diazepam (0.5 mg/kg) PO (n=48) | Anterograde amnesia | Recall of pictures on the following day. | Amnesia: no statistically significant differences |

PO = oral route; RR = rectal route; IV = intravenous route; IM= intramuscular route

| Lindgren et al, 1979, Finland [57] | Double-blind, parallel | 145 (0-15 years old) | Premedication  Medical (surgery)  Operating room | A: Atropine (0.02 mg/kg) and pethidine (1 mg/kg) IM (n=49) | Atropine (0.02 mg/kg) plus: B: diazepam (0.25 mg/kg) IM (n=46) C:flunitrazepam (0.02 mg/kg) IM (n=50) | Anterograde amnesia | Recall of pictures at 2 and 24 h after the operation | Amnesia (children ≥ 5 years): At 2 h: A: 22% (n=25); B: 10% (n=20); C: 33% (n=20) (*P*>0.05);  At 24 h: A: 11% (n=25); B: 10% (n=20); C: 33% (n=20) (*P*>0.05). |
| --- | --- | --- | --- | --- | --- | --- | --- | --- |
| Richardson and Manford, 1979, UK [62] | Double-blind, parallel | 142 children (not specified) | Premedication  Medical (surgery)  Operating room | A: flunitrazepam (1-2 mg) PO (n=72) | B: diazepam (10-20 mg) PO (n=70) | Anterograde amnesia | Recall of events, at the following day | Amnesia concerning induction: A: 74% (46/62); B: 39% (19/49) (*P*<0.001);  Amnesia concerning post-operative events: A: 81 % (38/47); B 49% (21/43) (*P*<0.01). |
