# Supplementary material for: What is the level of evidence for the amnestic effects of sedatives in pediatric patients? A systematic review and meta-analyses
Source: PLoS One. 2017 Jul 7;12(7):e0180248. doi: 10.1371/journal.pone.0180248 (PMC5501513; doi:10.1371/journal.pone.0180248)
Supplement: S4 Table — (DOC) [file pone.0180248.s006.doc]

# Amnestic effects: comparisons between benzodiazepines and non-benzodiazepine sedatives

| **Author, year, country** | **Study design** | **Participants**  **n (age)** | **Sedative use, procedure and setting** | **Sedative regimen** | | **Type of amnesia** | **Outcome measure** | **Outcome result/conclusion** |
| --- | --- | --- | --- | --- | --- | --- | --- | --- |
|  |  |  |  | **Intervention** | **Comparison** |  |  |  |
| Wilson et al, 2007, UK [51] | Open-label, crossover | 36 (10–15 years old) | Procedural sedation  Dental (extraction)  Outpatient | A: midazolam (0.2 mg/kg) MB (n=36) | B: nitrous oxide 30% (n=36) | Anterograde amnesia | Questionnaire about events, completed on the following day | Amnesia: greater in group A than in group B (*P*=0.289). |
| Koirala et al, 2006, Nepal [44] | Double-blind, parallel | 120 (2-9 years old) | Procedural sedation  Dental (not specified)  Outpatient | A: midazolam (0.5 mg/kg) PO (n=20) | B: ketamine (5 mg/kg) PO (n=20) C: zolpidem (0.4 mg/kg) PO (n=20) D: midazolam (0.4 mg/kg) + ketamine (3 mg/kg) PO (n=20) E: midazolam (0.5 mg/kg) + tramadol (2 mg/kg) PO (n=20) F: zolpidem (0.4 mg/kg) + tramadol (2 mg/kg) PO (n=20) | Anterograde amnesia | Questionnaire about events. | Amnesia: greater in the groups receiving drugs other than zolpidem. |
| Wilson et al, 2006, UK [50] | Open-label, crossover | 35 (5-10 years old) | Procedural sedation  Dental (extraction)  Outpatient | A: midazolam (0.3 mg/kg) PO (n=35) | B: nitrous oxide 30% (n=35) | Anterograde amnesia | Questionnaire about events, completed on the following day | Amnesia: greater in group A than in group B (*P*=0.031) |

| Singh et al., 2005, India [71] | Double-blind, parallel | 60 (2-10 years old) | Premedication  Not specified (surgery)  Operating room | A: midazolam (0.5 mg/kg) PO (n=30) | B: butorphanol (0.2 mg/kg) PO (n=30) | Anterograde amnesia | Questionnaire about events, applied postoperatively | Complete amnesia: A: 50% (15/30); B: 80% (24/30) (*P* <0.05) |
| --- | --- | --- | --- | --- | --- | --- | --- | --- |
| Singh et al, 2002, India [48] | Double-blind, parallel | 90 (3-9 years old) | Procedural sedation  Dental (extraction and restorative)  Outpatient | A: midazolam (0.5 mg/kg) PO (n=30) | B: triclofos (70 mg/kg) PO (n=30)  C: promethazine (1.2 mg/kg) PO (n=30) | Anterograde amnesia | Questionnaire about events, applied after 24 h | Amnesia: A: 70%; B: 30%; C: 10% |
| Funk et al, 2000, Germany [70] | Double-blind, parallel | 120 (2-10 years old) | Premedication  Medical and dental (surgery)  Operating room | A: midazolam (0.5 mg/kg) PO + ketamine (3 mg/kg) PO (n=39) | B: midazolam (0.5 mg/kg) PO (n=38) C: ketamine (6 mg/kg) PO (n=36) | Anterograde amnesia | Questionnaire about events, responded by parents at 1 and 7 days after surgery. | Complete amnesia at day 1: A: 49 % (19/39); B: 55% (21/38); C:39% (14/36)  Negative memories: At day 1: A: 5% (2/39); B: 5% (2/38); C: 14% (5/36).  At day 7: A: 3% (1/39); B: 11% (4/38); C: 8% (3/36). |
| Kupietzky et al, 1996, Israel [45] | Single-blind, parallel | 21 (24- 48 months old) | Procedural sedation  Dental (restorative)  Outpatient | A: hydroxyzine (3.7 mg/kg) PO (n=21) | B: midazolam (0.2 mg/kg) IN (n=21) | Anterograde amnesia | Recall of picture, at ten minutes after finishing the procedure | Amnesia: A: 19% (4/21); B: 67% (14/21) (*P*< 0.05). |
| Peters and Brunton, 1982, UK [60] | Double-blind, parallel | 199 children (≥1 year old) | Premedication  Medical (surgery)  Operating room | A: lorazepam (0.05 mg/kg) PO (n=112) | B: trimeprazine (3 mg/kg) PO (n=87) | Anterograde and retrograde amnesia | Recall of events pre- and post-sedative administration, on the following day | Anterograde amnesia: Group B showed less amnesia about venipuncture (9% vs 20%; *P*=0.03) and pain (49% vs 54%; *P*>0.05).  Retrograde amnesia: A: 9%; B: 9% (*P*>0.05) |

| Gordon and Turner, 1969, UK [56] | Double-blind, parallel | 161 (3-12 years old) | Premedication  Medical (surgery)  Operating room | A: hyoscine (0.4-0.6 mg) PO + phenobarbitone (4.4 mg/kg) PO (n=53) | Hyoscine (0.4-0.6 mg) PO plus: B: trimeprazine (3.3 mg/kg) (n=52) C: diazepam (0.22 mg/kg) (n=56) | Anterograde amnesia | Question about events, on the following day | Amnesia: A: 54, 5%; B: 54%; C: 66%. |
| --- | --- | --- | --- | --- | --- | --- | --- | --- |

PO = oral route; MB = buccal route; IN = intranasal route
