# Supplementary material for: What is the level of evidence for the amnestic effects of sedatives in pediatric patients? A systematic review and meta-analyses
Source: PLoS One. 2017 Jul 7;12(7):e0180248. doi: 10.1371/journal.pone.0180248 (PMC5501513; doi:10.1371/journal.pone.0180248)
Supplement: S5 Table — (DOC) [file pone.0180248.s007.doc]

# Amnestic effects: comparisons between benzodiazepines in combination with other drugs and any sedative

| **Author, year, country** | **Study design** | **Participants**  **n (age)** | **Sedative use, procedure and setting** | **Sedative regimen** | | **Type of amnesia** | **Outcome measure** | **Outcome result/conclusion** |
| --- | --- | --- | --- | --- | --- | --- | --- | --- |
|  |  |  |  | **Intervention** | **Comparison** |  |  |  |
| Sienkiewicz et al, 2015, Poland [47] | Blind, parallel | 48 (9–16 years old) | Procedural sedation  Medical (endoscopy procedures)  Operating room | A: Premedication: midazolam (0.3 mg/kg) PO.  Induction IV: atropine (10 μg/kg) + alfentanyl (5 μg/kg) + lignocaine (0.5 mg/kg) + midazolam (0.025 mg/kg). Maintenance: propofol (≤ 3 mg/kg/3 min) IV (n=26) | B: Premedication: midazolam (0.3 mg/kg) PO. Induction IV: atropine (10 μg/kg) + alfentanyl (5 μg/kg) + lignocaine (0.5 mg/kg) + midazolam (0.025 mg/kg). Maintenance: with midazolam (≤ 0.4 mg/kg) IV (n=25) | Anterograde amnesia | Questionnaire about procedural recall, at 30, 60 and 120 min after finishing endoscopy | Full amnesia: A: 25/26; B: 15/22 (Relative Risk of lack of amnesia B/A: 0.4, 95% CI 0.21 to 0.59). |
| Lee-Jayaram et al, 2010, USA [15] | Blind, parallel | 23 (5-17 years old) | Procedural sedation  Medical (fracture reduction)  Outpatient | A: ketamine (1 mg/kg) IV + midazolam (0.05 mg/kg) IV (n=11) | B: etomidate (0.2 mg/kg) IV + fentanyl (1 μg/kg) IV (n=12) | Anterograde amnesia | Questionnaire about procedural recall, before discharge | Recall: A: 0; B: 8% (1/12) (*P*>0.05) |

| Isik et al, 2008, Turkey [42] | Double-blind, parallel | 60 (3-8 years old) | Procedural sedation  Dental (not specified)  Outpatient | A: melatonin (3 mg) PO + nitrous oxide 40% (n=15) | Nitrous oxide 40% plus:  B: melatonin (0.5 mg/kg) PO (n=15) C: midazolam (0.75 mg/kg) PO (n=15) D: NaCl solution (n=15) | Anterograde amnesia | Observation of side effects | Amnesia: A: 0; B: 0; D: 0; C: 40% (6/15). |
| --- | --- | --- | --- | --- | --- | --- | --- | --- |
| Godambe et al, 2003, USA [14] | Blind, parallel | 113 (3-18 years old) | Procedural sedation  Medical (orthopedic procedures)  Outpatient | A: fentanyl (1–2 μg/kg) + propofol (1 mg/kg) IV (n=59) | B: midazolam (0.05 mg/kg) + ketamine (1-2 mg/kg) IV (n=54) | Anterograde amnesia | Recall of the procedure on return to baseline status | None of the subjects had any recall of the procedure (*P*=1). |
| Sullivan et al, 2001, USA [37] | Double-blind, crossover | 25 (30-66 months old) | Procedural sedation  Dental (restorative)  Outpatient | A: ketamine (8 mg/kg) and diazepam (0.1 mg/kg) PO (n=25) | B: ketamine (10 mg/kg) and diazepam (0.1 mg/kg) PO (n=25) | Anterograde and retrograde amnesia | Recall of a smell and the location of a box at the next appointment. | Anterograde amnesia: A: 78% (7/9); B: 56% (9/16) (*P*>0.05)  Retrograde amnesia: smell: A: 33% (3/9); B: 50% (8/16); location of a box: A: 22% (2/9); B: 6% (1/16) (*P*>0.05) |
| Auden et al, 2000, USA [26] | Double-blind, parallel | 51 (9 months-10 years old) | Premedication  Medical (catheterization)  Operating room | A: ketamine (6-10 mg/kg) + midazolam (0.6- 1 mg/kg) PO (n=23) | B: meperidine (2 mg/kg) + promethazine (1 mg/kg) + chlorpromazine (1 mg/kg) IM (n=28) | Anterograde amnesia | Recall of intraoperative events (children ≥ 4 years old), after finishing the procedure | Recall: A= 7% (1/14); B = 59% (10/17) (*P*= 0.007) |
| Kennedy et al, 1998, USA [34] | Open-label, parallel | 260 (5-10 years old) | Procedural sedation  Medical (painful procedure)  Outpatient | A: midazolam (≤ 0.3 mg/kg) IV+ ketamine (≤ 2 mg/kg) IV (n=130) | B: midazolam (≤ 0.3 mg/kg) IV + fentanyl (≤ 2 μg/kg) IV (n=130) | Anterograde amnesia | Recall about the procedure, before discharge | Complete amnesia: A: 87% (109/126); B: 85% (104/122) (*P*=0.85). |
| Marx et al, 1997, USA [16] | Double-blind, crossover | 18 ( 24-178 months old) | Premedication  Medical (painful procedures)  Outpatient | A: meperidine (2 mg/kg) IV + midazolam (0.1 mg/kg) (n=18) | B: atropine (0.01 mg/kg) IV + midazolam (0.05 mg/kg) + ketamine (1.5 mg/kg) (n=18) | Anterograde amnesia | Questions about adverse reactions, at 24 h and 7 days after the procedure. | Amnesia: A: 100% (15/15); B: 100% (15/15). |
| Bahal-O'Mara et al, 1994, USA [28] | Double-blind, parallel | 40 (1-17 years old) | Procedural sedation  Medical (endoscopy procedures)  Outpatient | A: meperidine (2 mg/kg) IV (n=21) | B: meperidine (2 mg/kg) + midazolam (0.05 mg/kg) IV (n=19) | Anterograde amnesia | Recall of a picture (children >3 years), immediately and at 24 h following the procedure. | Amnesia: Immediately following the procedure: A: 24% (4/17); B: 78% (14/18) (*P*= 0.002).  At a 24 h assessment: A: 24% (4/17); B: 75% (12/16) (*P*= 0.009). |

PO = oral route; IM= intramuscular route; IV = intravenous route
