# Supplementary material for: What is the level of evidence for the amnestic effects of sedatives in pediatric patients? A systematic review and meta-analyses
Source: PLoS One. 2017 Jul 7;12(7):e0180248. doi: 10.1371/journal.pone.0180248 (PMC5501513; doi:10.1371/journal.pone.0180248)
Supplement: S6 Table — (DOC) [file pone.0180248.s008.doc]

Amnestic effects: comparisons of non-benzodiazepine drugs and non-benzodiazepine drugs

| **Author, year, country** | **Study design** | **Participants**  **n (age)** | **Sedative use, procedure and setting** | **Sedative regimen** | | **Type of amnesia** | **Outcome measure** | **Outcome result/conclusion** |
| --- | --- | --- | --- | --- | --- | --- | --- | --- |
|  |  |  |  | **Intervention** | **Comparison** |  |  |  |
| Singh et al, 2014, India [49] | Triple-blind, parallel | 112 (3-10 years old) | Procedural sedation  Dental (not specified)  Outpatient | A: ketamine (8 mg/kg) PO (n=28) | B: dexmedetomidine (3 μg/kg) PO (n=28) C: dexmedetomidine (4 μg/kg) PO (n=28)  D: dexmedetomidine (5 μg/kg) PO (n=28) | Anterograde amnesia | Recall of images and events, at the postoperative assessment | Amnesia concerning images: A: 64 % (18/28); B: 18 % (05/28); C: 36%(10/28); D: 43% (12/28) (*P*<0.01);  Amnesia concerning events: A: 61% (17/28); B: 21% (06/28); C: 25% (07/28); D: 39% (11/28) (*P*<0.01). |
| Ghadami Yazdi et al, 2013, Iran [13] | Double-blind, parallel | 60 (3-12 years old) | Procedural sedation  Medical (puncture or aspiration)  Outpatient | A: ketamine (25 mg) + propofol (50 mg) IV (n=30) | B: ketamine (17 mg) + propofol (50 mg) IV (n=30) | Anterograde amnesia | Not specified | None of the children had a bad memory |
| Yun, 2003, Korea [65] | Double-blind, parallel | 60 (5-12 years old) | Premedication  Medical (surgery)  Operating room | A: clonidine (1 μg/kg) PO (n=20) | B: clonidine (2 μg/kg) PO (n=20); C: saline (n=20) | Anterograde amnesia | Questionnaire about memory of procedure, responded immediately after arrival in the recovery room. | Amnesia: A: 30%; B: 55%; C: 15% (*P*<0.05 for B-A and B-C) |
| Lembert et al, 2002, France [46] | Double-blind, parallel | 48 children (≥5 years old) | Procedural sedation  Medical (propofol injection)  Operating room | A: nitrous oxide 50% + propofol (4 mg/kg) IV (n=24) | B: propofol (4 mg/kg) IV + lidocaine (0.2 mg/kg) (n=24) | Anterograde amnesia | Memory of pain, VAS | Recall: A: 0% (0/27) scored a VAS>4; B: 30% (7/23) scored a VAS>4 (*P*<0.05) |

| Zsigmond et al, 1996, Hungary [17] | Open-label, parallel | 30 (1-6 years old) | Premedication  Medical (surgery)  Outpatient | A: ketamine (6.0 mg/kg) jet-injection (j.i) and atropine (20 μg/kg) (n=10) | Atropine (20 μg/kg) plus  B: ketamine (3.5 mg/kg) j.i. (n=10)  C: ketamine (2.5 mg/kg) j.i (n=10) | Anterograde amnesia | Recall of events and pain at the follow-up. | Complete amnesia was present in all patients. |
| --- | --- | --- | --- | --- | --- | --- | --- | --- |
| Evans et al., 1995, USA [30] | Open-label, parallel | 30 (4-15 years old) | Procedural sedation  Medical (reduction of fracture)  Outpatient | A: nitrous oxide 50% (n=15) | B: meperidine (2 mg/kg) IM + promethazine (1 mg/kg) IM (n=15) | Anterograde amnesia | Questionnaire about memory of the procedure, responded at the first follow-up visit. Scores ranged from 0 (everything) to 5 (nothing). | Memory score: A: mean 2.2; B: 1.2 (*P*>0.05) |
| Terndrup et al, 1993, USA [38] | Double-blind, parallel | 87 (≤16 years old) | Procedural sedation  Medical ( emergency procedures)  Outpatient | A: meperidine (2 mg/kg) IM +promethazine (1 mg/kg) IM (n=43) | B: meperidine (2 mg/kg) IM + promethazine (1 mg/kg) + chlorpromazine (1 mg/kg) n=44) | Anterograde amnesia | Questionnaire about delayed complications, including bad memories | Bad memories: A: 7% (3/44); B: 7% (3/43) |

PO = oral route; IM= intramuscular route; IV = intravenous route; VAS= visual analogic scale
